# Low-Coordinate Erbium(III) Single-Molecule Magnets with Photochromic Behavior

**DOI:** 10.1021/acs.inorgchem.2c01999

**Published:** 2022-10-05

**Authors:** Katarzyna Rogacz, Maria Brzozowska, Sebastian Baś, Katarzyna Kurpiewska, Dawid Pinkowicz

**Affiliations:** Faculty of Chemistry, Jagiellonian University, Gronostajowa 2, 30-387Kraków, Poland

## Abstract

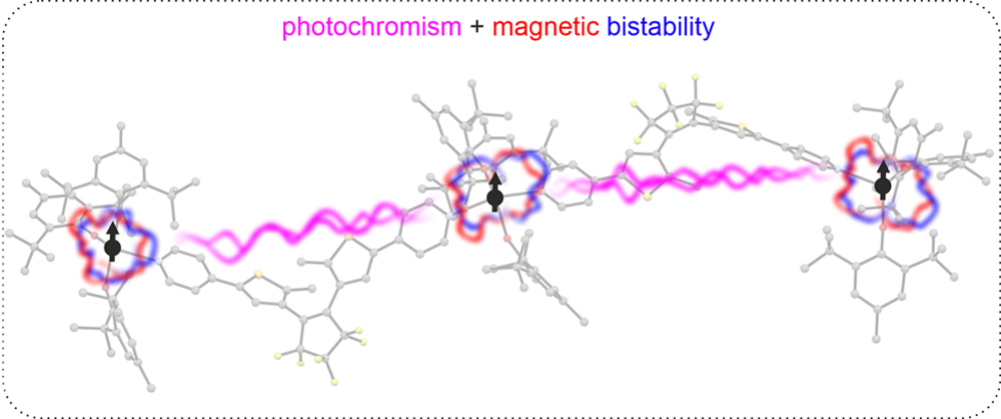

The structures and magnetic properties of photoresponsive magnets can be controlled or fine-tuned by visible light irradiation, which makes them appealing as candidates for ternary memory devices: photochromic and photomagnetic at the same time. One of the strategies for photoresponsive magnetic systems is the use of photochromic/photoswitchable molecules coordinated to paramagnetic metal centers to indirectly influence their magnetic properties. Herein, we present two erbium(III)-based coordination systems: a trinuclear molecule {[Er^III^(BHT)_3_]_3_(dtepy)_2_}^.^4C_5_H_12_ (**1**) and a 1D coordination chain {[Er^III^(BHT)_3_(azopy)}_*n*_·2C_5_H_12_ (**2**), where the bridging photochromic ligands belong to the class of diarylethenes: 1,2-bis((2-methyl-5-pyridyl)thie-3-yl)perfluorocyclopentene (dtepy) and 4,4′-azopyridine (azopy), respectively (BHT = 2,6-di-*tert*-butyl-4-methylphenolate). Both compounds show slow dynamics of magnetization, typical for single-molecule magnets (SMMs) as revealed by alternating current (AC) magnetic susceptibility measurements. The trinuclear compound **1** also shows an immediate color change from yellow to dark blue in response to near-UV irradiation. Such behavior is typical for the photoisomerization of the open form of the ligand into its closed form. The color change can be reversed by exposing the closed form to visible light. The chain-like compound **2**, on the other hand, does not show significant signs of the expected *trans*–*cis* photoisomerization of the azopyridine in response to UV irradiation and does not appear to show photoswitching behavior.

## Abstract 2

Three-coordinate [Er^III^(BHT)_3_] single ion magnets undergo ligand addition reaction in pentane to form linear trinuclear photochromic nanomagnets where both functionalities persist.

## Introduction

Multifunctionality is one of the main goals and a great promise of materials science.^1−4^ It is also a hot topic in the subfield of molecular materials, which promises to deliver single molecules with multiple intertwined functions. The multifunctionality in molecule-based systems can be achieved by a rational design/choice of building blocks and their smart assembly into various classes of molecules and molecular solids ranging from purely organic compounds and mononuclear coordination complexes to sophisticated multidimensional polymeric structures including coordination polymers. One of the many classes of multifunctional molecule-based compounds are magnets responsive to visible light—molecular photomagnets.^5,6^ Photomagnets enable the control of magnetic properties by photons^7^—a feature that can be achieved by design in molecular systems but is hardy accessible in conventional magnetic materials except some orthoferrites^8^ and the transparent films of some garnets.^9^

One of the strategies toward photomagnetic compounds is the incorporation of photochromic molecules widely used by Feringa et al. to achieve unique photomechanical properties^10,11^ and by Lehn et al. for photo- and electroswitchable molecular devices.^12^ Such molecules coordinated to paramagnetic centers enable the control of the magnetic properties of the resulting compound upon reversible photoisomerization reaction of the ligand.^13−16^ The control occurs through the modification of the magnetic interaction pathways (the two photoisomers need to transmit these interactions differently)^17^ or by influencing the coordination geometry of the paramagnetic species. Dithienylethenes^18^ (a subgroup of diarylethenes^19^) seem to be the most widely used photochromic ligands for the construction of photomagnetic assemblies.^20^ Some of the current trends in the field of molecular photomagnets focus on the incorporation of the photoswitching functionality into lanthanide single-molecule magnets (Ln-SMMs)—compounds that show magnetization blocking below a certain blocking temperature *T*_B_.^21−23^ The use of photochromic ligands in the construction of Ln-SMMs^15,16,24^ allows the control of various magnetization relaxation mechanisms—usually the quantum tunneling of magnetization.^16^ However, the main targets are the temperature-dependent relaxation mechanisms: Raman and Orbach. To the best of our knowledge, all photochromic Ln-SMM systems are based on Ln centers with high coordination numbers, which make their design challenging and serendipitous according to the Ln-SMM design strategies proposed by Tong and co-workers.^25^ Herein, we take an approach where a low-coordinate lanthanide complex showing SMM behavior due to the favorable geometry—in this case, the Er^III^ complex with three phenolate-type ligands located in the equatorial positions—is approached only by one or two monodentate weak-field ligands so that the SMM character is retained and enriched by an additional functionality—photoswitching. This strategy was proposed by some of us previously and demonstrated by the substitution of THF by the TEMPO molecule in the structure of [Er^III^(BHT)_3_(THF)]^26^ (BHT = 2,6-di-*tert*-butyl-4-methylphenolate). In this work particularly, the four-coordinate [Er^III^(BHT)_3_(THF)]^26^ and the three-coordinate [Er^III^(BHT)_3_]^27^ single-molecule magnets (SMMs) are reacted with two types of N-donor diarylethene-type bridging ligands that are well known to undergo photoisomerization: 1,2-bis((2-methyl-5-pyridyl)thie-3-yl)perfluorocyclopentene (dtepy)^28,29^ and 4,4′-azopyridine (azopy).^30,31^ The respective reactions yield two coordination species: the trinuclear linear {[Er^III^(BHT)_3_]_3_(dtepy)_2_}^.^4C_5_H_12_ complex (**1**) and a coordination chain {[Er^III^(BHT)_3_(azopy)·2C_5_H_12_}_*n*_ (**2**). The reaction takes place in a strictly non-coordinating solvent, *n*-pentane, with a strictly controlled number of bridging ligands. Hence, the coordination number of the Er^III^ centers increases in a controlled manner from 3 to 4 and 5 in compound **1** and from 3 to 5 in compound **2**. As aforementioned, this strategy enables the retention of the SMM functionality and the observation of the photoisomerization associated with dtepy ligand in compound **1**, while compound **2** remains unresponsive to light, most probably due to the more rigid chain structure. The combination of both functionalities: the SMM-type slow magnetic relaxation and the reversible ligand photoisomerization are presented and discussed.^32^

## Results and Discussion

### Synthesis

**Figure 1:**
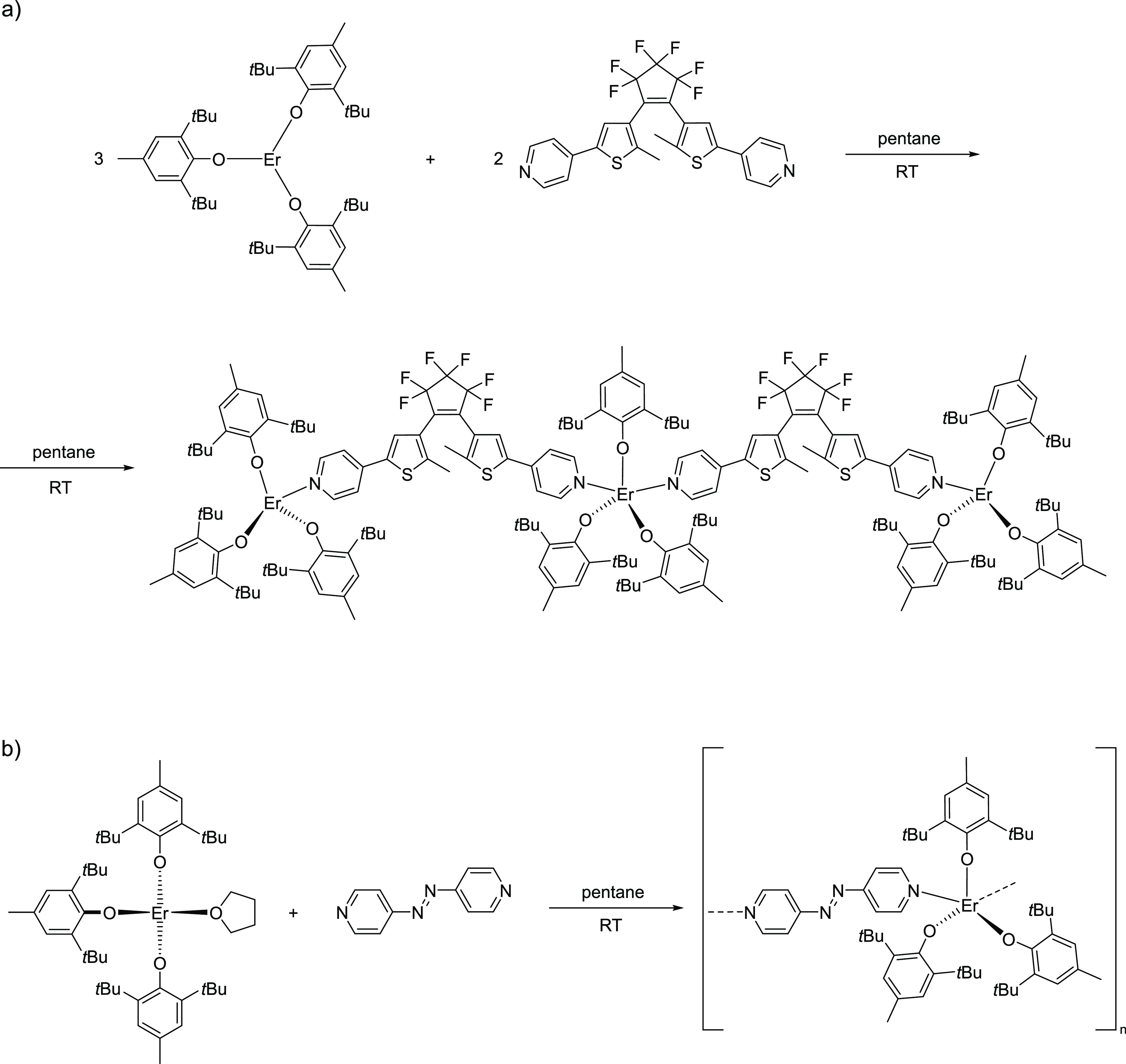
Diagrams showing the preparation strategy of compound **1** (a) and compound **2** (b) from the respective building blocks.

**Figure 2:**
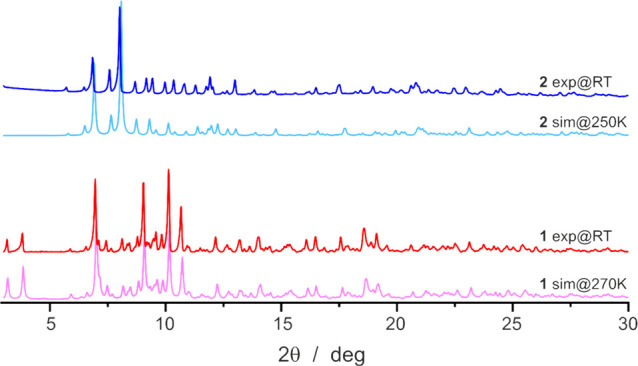
Comparison of the experimental PXRD for compound **1** (red line) and compound **2** (blue line) with the simulated ones obtained from scXRD data collected at 270 K for **1** (pink line) and at 250 K for **2** (light blue line). Slight differences for **2** are caused by a larger temperature difference between both measurements.

The reported compounds are obtained in one-pot reactions where the respective substrates—the Er^III^ complex and the ligand—are combined in pentane, stirred at room temperature for a few minutes, and left for spontaneous crystallization overnight (Figure 1). {[Er^III^(BHT)_3_]_3_(dtepy)_2_}^.^4C_5_H_12_ (**1**) crystallizes in the form of small yellow prism crystals that change color to greenish-blue when exposed to the LED light of the stereomicroscope. {[Er^III^(BHT)_3_(azopy)}_*n*_·2C_5_H_12_ (**2**), on the other hand, forms larger purple/violet crystals that do not appear to be photochromic. The preparation of both compounds requires strict control of the reaction stoichiometry. Any deviation from the provided procedure (see the Experimental Section for details) leads to by-products, which are visible as additional reflections in the powder X-ray diffraction (PXRD) experiments. These by-products could not be identified at this point. If the reaction is followed to the letter, the experimental PXRD patterns recorded at room temperature are identical with those simulated from the single-crystal X-ray diffraction (scXRD) obtained at 270 K (Figure 2).

### Single-Crystal X-ray Diffraction Structures

#### Crystal Structure of {[Er^III^(BHT)_3_]_3_(dtepy)_2_}^.^4C_5_H_12_ (**1**)

**Figure 3:**
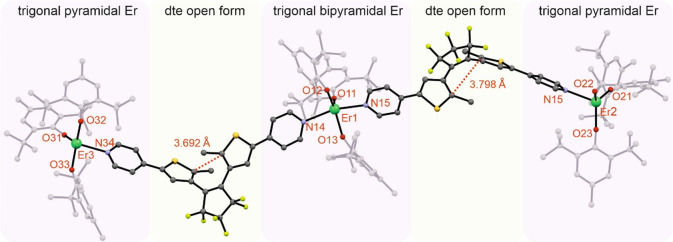
Diagram showing the scXRD structure of compound **1** (Er, green; S, yellow; C, gray; N, blue; F, greenish-yellow; H, omitted for clarity). The colored panels highlight different parts of the trinuclear molecule: [Er^III^(BHT)_3_] units are pink, and bridging dtepy ligands in the open form are yellow. The indicated C···C distances of 3.692 and 3.798 Å are within the proper range for the photocyclization reaction to occur even in the solid state (shorter than the 4.2 Å limit).^33^

{[Er^III^(BHT)_3_]_3_(dtepy)_2_}^.^4C_5_H_12_ (**1**) crystallizes at room temperature from the pentane mother solution in the monoclinic space group *P*2_1_/c with the whole linear trinuclear molecule in the asymmetric unit (Figure 3). Selected crystallographic parameters are gathered in Table S1 in the SI.

Due to the linear geometry, there are two types of Er^III^ centers in the molecule: two terminal and one central. The two terminal ones are four-coordinate and adopt a distorted trigonal pyramidal geometry with three O-coordinated BHT and one N-coordinated dtepy ligand. The central cation is five-coordinate with three O-coordinated BHT ligands in the equatorial plane and two N-coordinated dtepy ligands occupying the axial positions of the distorted trigonal bipyramid. The dtepy molecules act as bridging ligands connecting the terminal Er^III^ ions with the central one via the nitrogen atoms of the pyridine groups. The intramolecular distances between the central and terminal Er^III^ ions are 19.357(1) and 19.597(1) Å. The distance between the two photoreactive carbon atoms of the thiophene rings are within the 3.7–3.8 Å range as indicated in Figure 3, which, according to the literature reports, enables the photocyclization reaction to occur even in the solid state.^33^

Table 1 gathers selected bond lengths and angles of the coordination spheres of the Er^III^ ions. The Er–O bond lengths in the case of the terminal erbium atoms are within the 2.056(5)–2.094(4) Å range, which is similar to the previous reports for lanthanide–aryloxide complexes.^26,27,34^ Of note, the analogous distances for the central erbium are slightly longer (2.095(5)–2.145(5) Å). Similar elongation can be observed for Er–N bonds: the terminal units show Er–N of 2.459(6)–2.461(5), while the central ones are longer (2.499(6)–2.519(5)). The distortion from the ideal trigonal pyramidal geometry is caused mainly by unusually large O–Er–O angles 125.22(17)° for O32–Er3–O33 and 122.37(19)° for O21–Er2–O22. As aforementioned, the bipyramidal geometry of the central Er^III^ is also distorted. The N–Er–N angle deviates from 180° by ca. 30°, and the O–Er–O angles are larger than the expected 120° (i.e., 136.04(18)° for O13–Er1–O12).

**Table 1:** Bond Lengths and Angles of the Coordination Spheres of Er^III^ Ions in Compound **1**

	central Er^III^(*x* = 1)	“right”^a^ terminal Er^III^(*x* = 2)	“left”^a^ terminal Er^III^(*x* = 3)
bond lengths (Å)			
Erx–Ox1	2.095(5)	2.056(5)	2.056(4)
Erx–Ox2	2.145(5)	2.094(4)	2.089(4)
Erx–Ox3	2.142(5)	2.089(5)	2.085(4)
Erx–Nx1	2.519(5)	2.459(6)	2.461(5)
Erx–Nx2	2.499(6)		
angles (°)			
Ox1–Erx–Ox2	112.98(19)	122.37(18)	109.79(17)
Ox1–Erx–Ox3	110.98(19)	107.51(18)	118.24(17)
Ox3–Erx–Ox2	136.04(18)	122.34(18)	125.22(17)
Ox1–Erx–Nx4	104.3(2)	105.67(19)	109.17(18)
Ox3–Erx–Nx4	84.32(19)	109.49(18)	87.60(18)
Ox2–Erx–Nx4	84.66(19)	84.43(19)	100.62(17)
Ox1–Erx–Nx5	107.3(2)		
Ox3–Erx–Nx5	82.55(19)		
Ox2–Erx–Nx5	85.0(2)		
Nx5–Erx–Nx4	148.28(19)		

a “Right” and “left” refer to the positions of the terminal Er^III^ ions as shown in Figure 3.

The shortest distance between the two Er^III^ centers located in the adjacent molecules (the intermolecular Er···Er distance) is 13.438(1) Å. Figure S1 presents the packing diagram of the Dy-dtepy-Dy-dtepy-Dy cores, with the shortest intermolecular distances highlighted as a dotted line. Such a long through-space distance between the paramagnetic centers and even longer intramolecular separation through the dtepy ligands suggest that magnetic interactions can be completely neglected in the analysis of the magnetic properties of **1**.

#### Crystal Structure of {[Er^III^(BHT)_3_(azopy)}_*n*_·2C_5_H_12_ (**2**)

**Figure 4:**
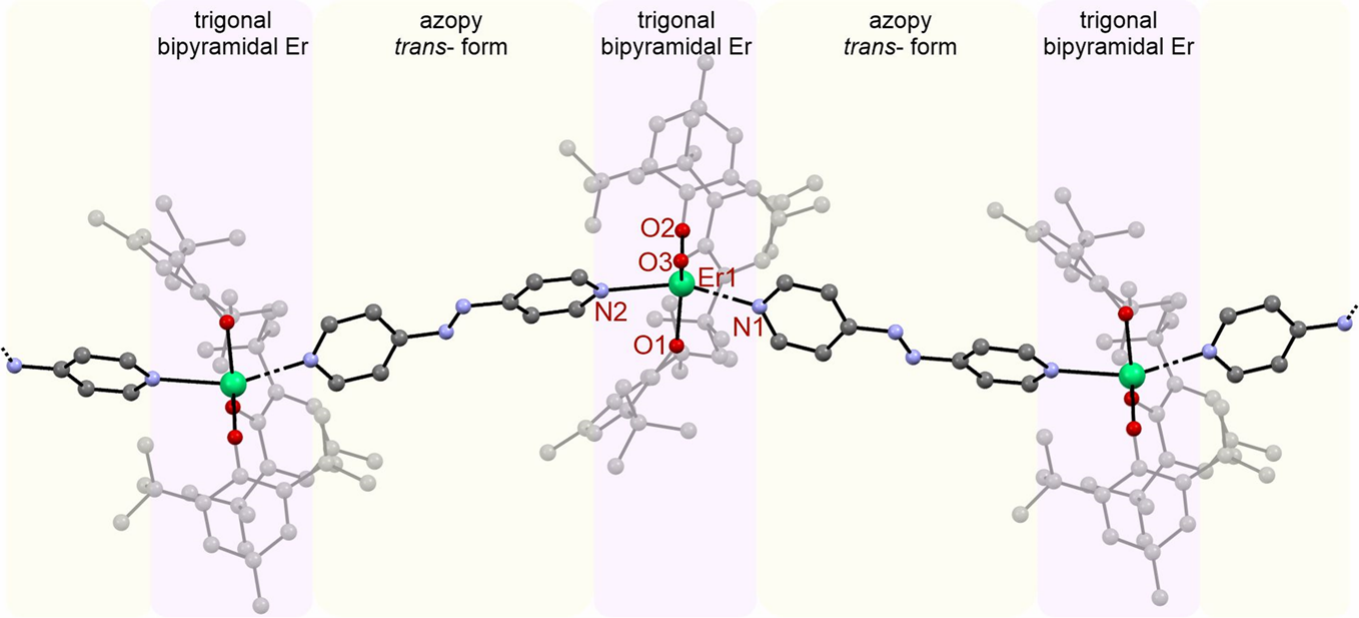
Diagram showing the scXRD structure of the coordination chain **2** (Er, green; C, gray; N, blue; H, omitted for clarity). The colored panels highlight different parts of the chain: Er^III^ trigonal bipyramidal units are pink, and bridging azopy ligands are yellow.

{[Er^III^(BHT)_3_(azopy)}_*n*_·2C_5_H_12_ crystalizes in the monoclinic crystal system in the *P*2_1_/c space group in forms of slightly elongated purple prisms as determined by scXRD structural analysis. Table S1 summarizes the crystallographic parameters for compound **2**. {[Er^III^(BHT)_3_(azopy)}_*n*_·2C_5_H_12_ forms slightly wavy coordination chains along the *b* crystallographic direction with the azopy acting as a bridging ligand connecting “Er^III^(BHT)_3_” units (Figure 4). The spaces between the chains are filled with pentane as the crystallization solvent.

Each coordination chain comprises five-coordinate Er^III^ ions of an approximate trigonal bipyramidal geometry and azopy ligands that play the role of molecular bridges. The Er^III^ centers are coordinated by three oxygen atoms of the three BHT ligands located in the equatorial plane and two nitrogen atoms in the approximately axial positions. The Er–O bond lengths (Table 2) are 2.154(3), 2.137(3), and 2.090(3) Å with an average value of 2.127(3) Å, similar to compound **1** and other reported lanthanide–arylozide complexes. The Er-N bonds are significantly longer due to the neutral character of the azopy ligands with values of 2.462(8) and 2.503(6) Å. Again, these values are similar to those discussed for compound **1**. The N–Er–N angle is 152.6(2)°, meaning that the trigonal bipyramid is noticeably bent from 180°. The length of the N3–N4 bond in the azopy molecular bridge is 1.227(8) Å, which is slightly smaller than the corresponding bond of the uncoordinated molecule 1.249(1) Å.^35^ This confirms that the N–N bond in the coordinated azopy is still a double bond. Interestingly, compound **2** exhibits orientational disorder of the azopy ligand, with ca. 10% mirrored along its long axis. This suggests pedal motions at room temperature, which become frozen during the flash cooling of the crystal in the cryostream; similar behavior was reported for the azobenzene molecule.^35^

**Table 2:** Bond Lengths and Angles within the Coordination Sphere of the Er^III^ Ion in Compound **2**

	bond length (Å)		angle (°)
Er–O1	2.155(3)	O1–Er–O2	137.9(1)
Er–O2	2.137(4)	O2–Er–O3	111.3(1)
Er–O3	2.090(4)	O3–Er–O1	110.6(1)
Er–N1	2.461(8)	O1–Er–N1	84.4(2)
Er–N2	2.503(6)	O2–Er–N1	85.4(2)
N3–N4	1.227(8)	O3–Er–N1	98.2(2)
		O1–Er–N2	85.7(2)
		O2–Er–N2	84.9(2)
		O3–Er–N2	109.2(2)
		N1–Er–N2	152.6(2)

The shortest interchain Er···Er distance is 11.040(1) Å, while the intrachain one is considerably longer at 13.938(1) Å. Figure S2 presents the packing diagram of the -(Er-azopy)- chains.

#### Photochromic Behavior of **1** and **2**

**Figure 5:**
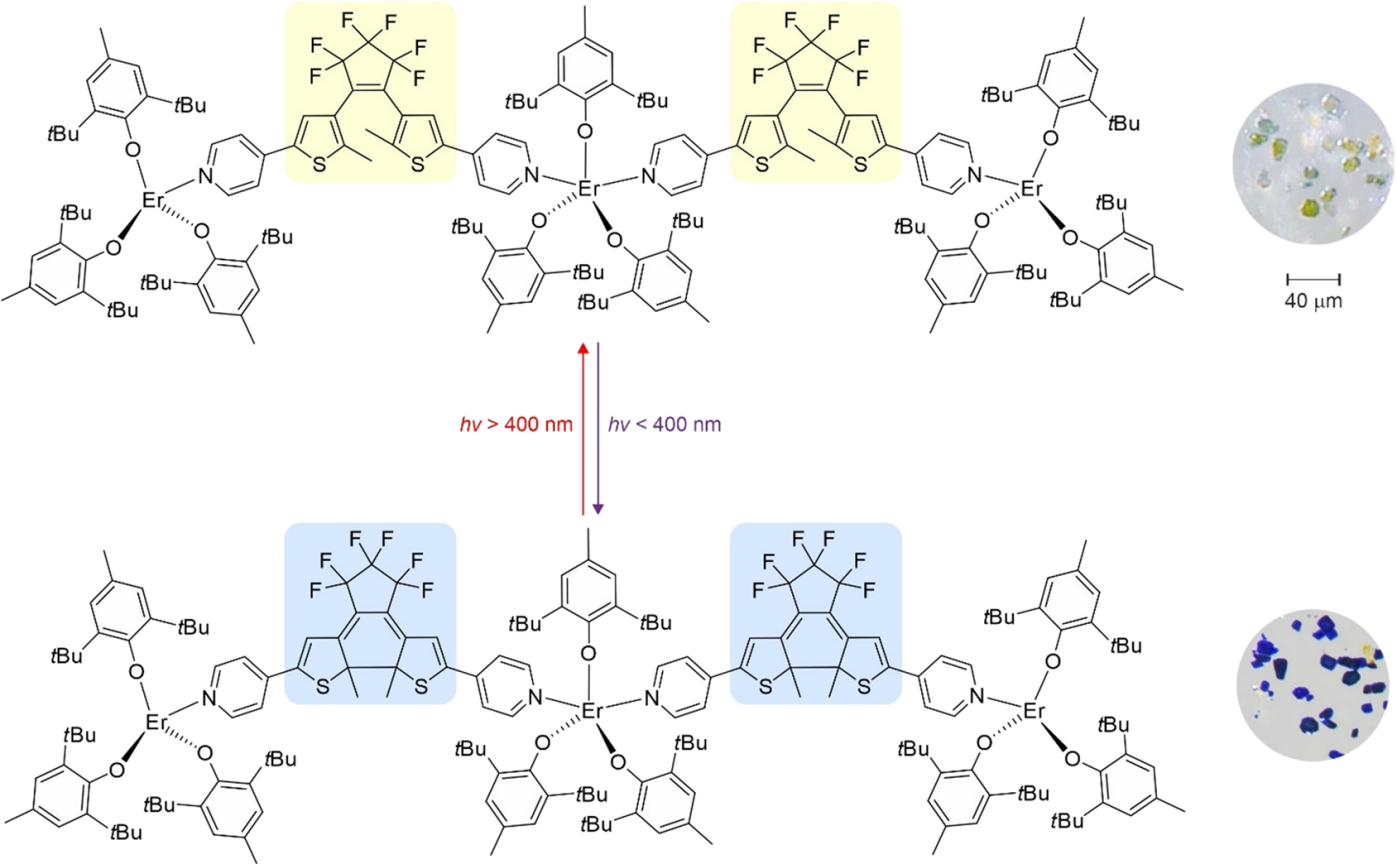
Diagram showing the photocyclization of dtepy ligands in compound **1** upon UV light irradiation, resulting in **1UV** (left), and photographs of the crystals of **1** before and after UV light irradiation (right). Please note that the crystals before irradiation are yellow with a bluish tinge due to the cold LED light of the microscope causing the photoisomerization.

As already mentioned in the previous section, compound **1** exhibits quite clear symptoms of photochromic behavior—cold white-light illumination leads to a distinct color change of the yellow crystals to blue (green at first) and dark blue, typical for this class of compounds (Figure 5).^36^ Compound **2**, on the other hand, does not show any color changes under similar conditions. Since photochromic behavior is expected in both cases, both compounds were investigated using UV–vis and IR spectroscopy before and after UV light irradiation (365 nm; **1UV**) and then, if the change is observed, after visible light irradiation (638 nm; **1vis**) to confirm the reversibility of the process. Compound **2** does not show any appreciable changes in the UV–vis and IR spectra after UV photoexcitation, while compound **1** exhibits clear changes, which are discussed in the following two subsections. Attempts to determine the crystal structure of the compound after UV light irradiation (**1UV**) using XRD techniques were unsuccessful—the compound loses long-range structural order; in other words, it does not show X-ray diffraction after photocyclization.

#### UV–Vis Spectroscopy

**Figure 6:**
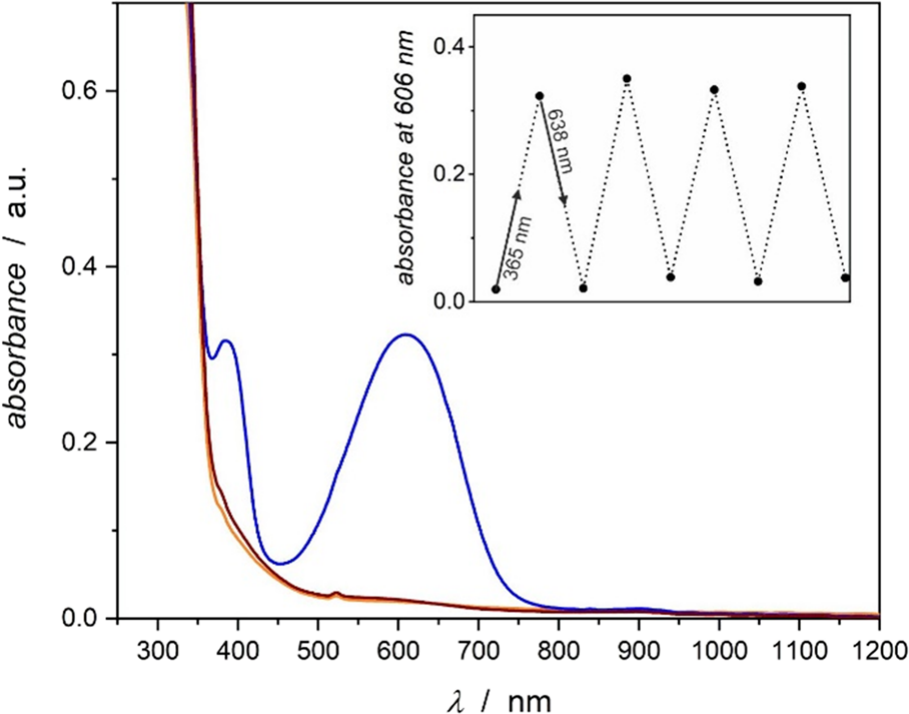
Photochromic behavior of **1** followed by UV–vis absorption spectroscopy in the solid state at room temperature (orange line, pristine compound **1** before irradiation; blue line, after 365 nm irradiation **1UV**; brown line, after 638 nm irradiation **1vis**). Inset: Changes of the absorbance at 606 nm in four consecutive dtepy ring closing and opening photoisomerization cycles.

Compound **1** is yellow, and its UV–vis absorption spectrum in the solid state is shown in Figure 6 (orange solid line). It shows absorption bands below 400 nm associated mostly with the open form of the dtepy. Upon 365 nm irradiation, within the lowest energy band of the open form, two new broad bands located at λ_max1_ = 606 nm and λ_max2_ = 380 nm appear; the compound becomes dark blue mostly due to the λ_max1_ absorption in the 550–650 nm range (Figure 6, blue solid line). The new bands are associated with the absorption of the closed form of the dtepy ligand in the compound denoted as **1UV**.^37^ The consecutive 638 nm light irradiation (red light) restores the original yellow color of **1vis** and its UV–vis spectrum is identical with that of the pristine **1** (Figure 6, brown solid line). The photochromic switching can be repeated in several cycles with the sample changing color between yellow and blue with no apparent photodegradation (Figure 6, inset)—a feature promising from the point of view of photoswitching of the magnetic properties.

#### IR Spectroscopy

**Figure 7:**
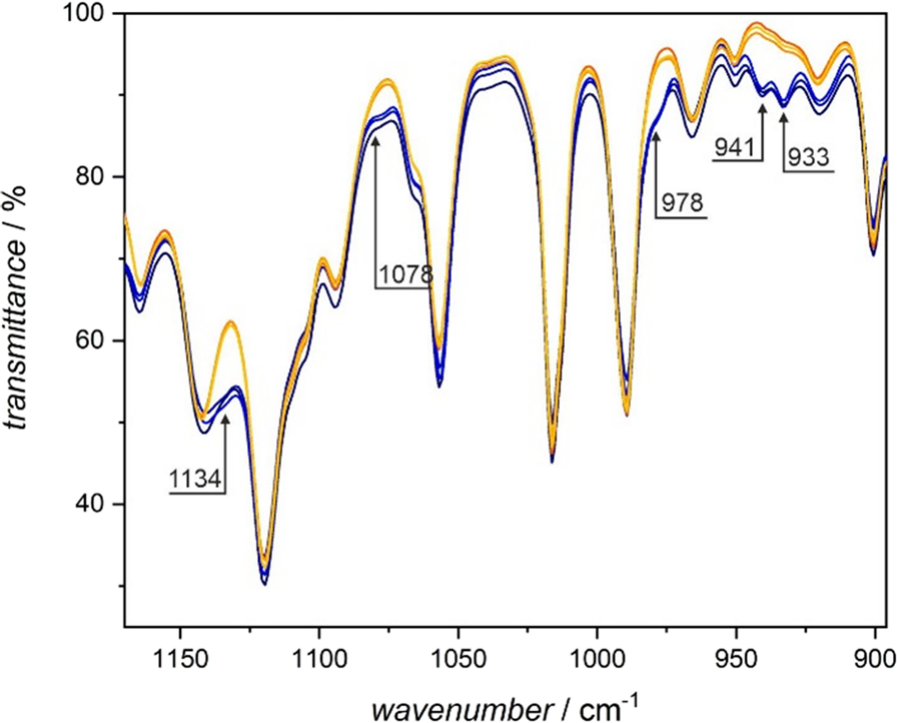
Photochromic behavior of **1** followed by IR spectroscopy in the solid state at room temperature in three consecutive photoswitching cycles (blue lines represent IR spectra after violet light irradiation while the three yellowish-orange ones correspond to the IR spectra of the open forms obtained after red light irradiation of the closed form). The violet light leads to photocyclization, which results in the appearance of additional bands at 1134, 1078, 978, 941, and 933 cm^–1^, as indicated in the plot with the ones at 941 and 933 cm^–1^, corresponding to the newly forming C–C bond between the thiophene rings upon photocyclization.

The photochromism of **1** can also be followed using IR spectroscopy. Figure 7 shows the fingerprint region of **1**. The photocyclization caused by violet light irradiation results in the appearance of additional bands associated with the formation of the central C_6_ ring in the closed form of the dtepy ligand. The consecutive red light irradiation (visible light) reverses the process, leading to an identical IR spectrum as before the experiment. The photoisomerization experiment was repeated three times as indicated in Figure 7.

### Magnetic Studies

#### DC Magnetic Properties of **1** and **2** before and after Irradiation

**Figure 8:**
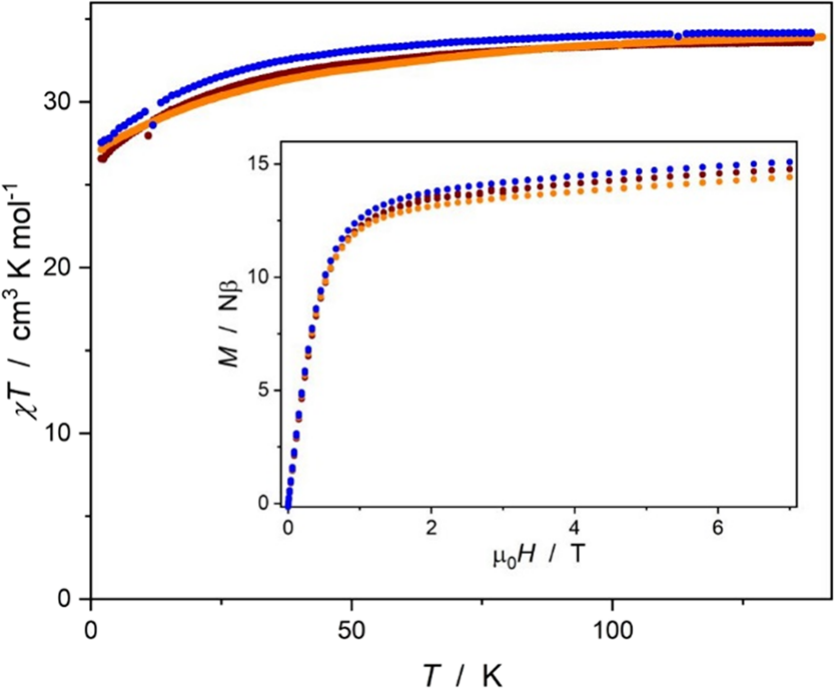
χ*T*(*T*) at 0.1 T (main) and *M*(*H*) (inset) at 1.8 K for **1** (orange points), **1UV** (blue points), and **1vis** (brown points). The experimental dependences for **1UV** corresponding to the closed form of the dtepy ligand are significantly shifted from those comprising its open form: **1** and **1vis**.

**Figure 9:**
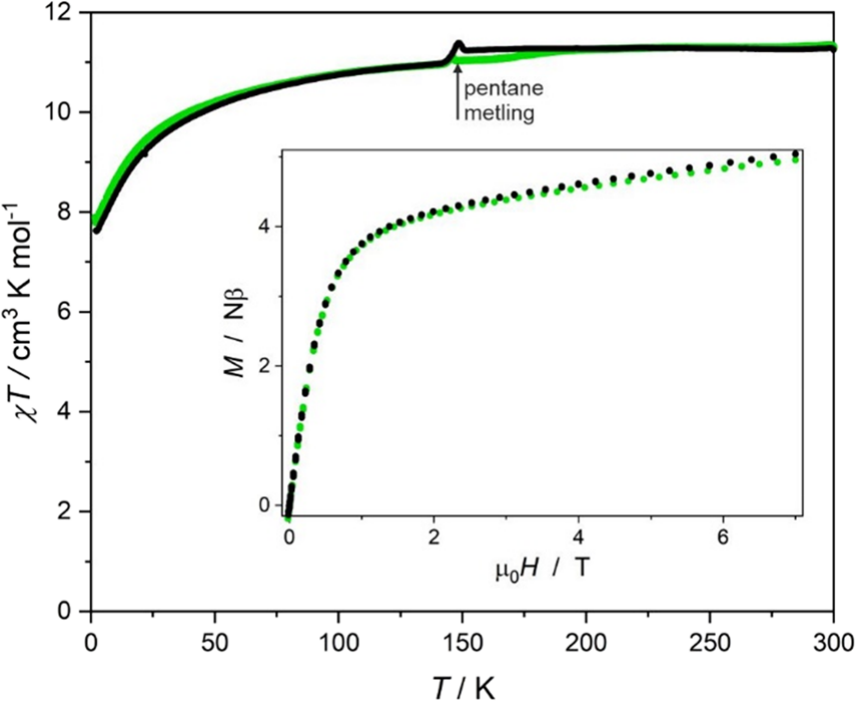
χ*T*(*T*) at 0.1 T (main) and *M*(*H*) (inset) at 1.8 K for **2** (green points) and **2UV** (black points). The anomaly at ca. 150 K is due to the melting of pentane, which covers the samples.

DC (direct current) magnetic measurements for **1**, **1UV**, and **1vis** as well as for **2** and **2UV** were performed using a SQUID magnetometer. For **1**, **1UV**, and **1vis**, the temperature dependence was recorded in the 2–140 K range (limited due to the significant movement of the crystallites above the freezing point of pentane) and the external DC field of 0.1 T (Figure 8), while for **2** and **2UV**, a typical 2–300 K range could be used (Figure 9; in this case, the movement of the crystallites was negligible). The magnetic field dependence at a constant temperature of 1.8 K was recorded in the 0–7 T range for both systems (insets in Figures 8 and 9).

In the case of **1**, **1UV**, and **1vis**, the temperature dependence of the molar magnetic susceptibility achieves a plateau in the 100–140 K range with values close to 34.4 cm^3^ K mol^–1^, expected for three non-interacting Er^III^ ions in the free-ion approximation (^4^I_15/2_, *g*_J_ = 6/5): 33.9 cm^3^ K mol^–1^ for **1**, 34.2 cm^3^ K mol^–1^ for **1UV**, and 33.7 cm^3^ K mol^–1^ for **1vis**. The χ*T*(*T*) gradually decreases on cooling and reaches 27.1 for **1**, 27.5 cm^3^ K mol^–1^ for **1UV**, and 26.5 cm^3^ K mol^–1^ for **1vis** at 1.8 K. Compound **2** and its UV irradiated form **2UV** exhibit very similar χ*T*(*T*) behavior. The observed χ*T*(*T*) decrease in both compounds in all states is ascribed to the thermal depopulation of the excited *m*_J_ sublevels. The χ*T*(*T*) traces for **1**, **1UV**, and **1vis** are very similar, which prevents any direct conclusions regarding the influence of the photocyclization of the ligand on the DC magnetic properties. Similarly, the χ*T*(*T*) for **2** and **2UV** overlap almost perfectly, indicating negligible influence of the UV irradiation on the DC magnetic properties of **2**, consistent with results of the UV–vis and IR experiments.

The *M*(*H*) curves are quite typical for Er^III^ complexes with approximate C_3_ symmetry.^38,39^ The curves for all three states of compound **1** and for the two states of **2** are nearly identical and differ only in the level of the quasi-plateau reflected by the maximum magnetization value at 7 T: 14.4 Nβ for **1**, 15.1 Nβ for **1UV**, 14.7 Nβ for **1vis**, 4.9 Nβ for **2**, and 5.0 Nβ for **2UV**. The changes in *M*(*H*) dependencies within the **1** → **1UV** → **1vis** sequence also seem to be very mild and cannot be unequivocally associated with the influence of the dtepy reversible photocyclization. The magnetic hysteresis could not be observed for any of the compounds even after irradiation.

#### AC Magnetic Properties of **1** and **2** before and after Irradiation

**Figure 10:**
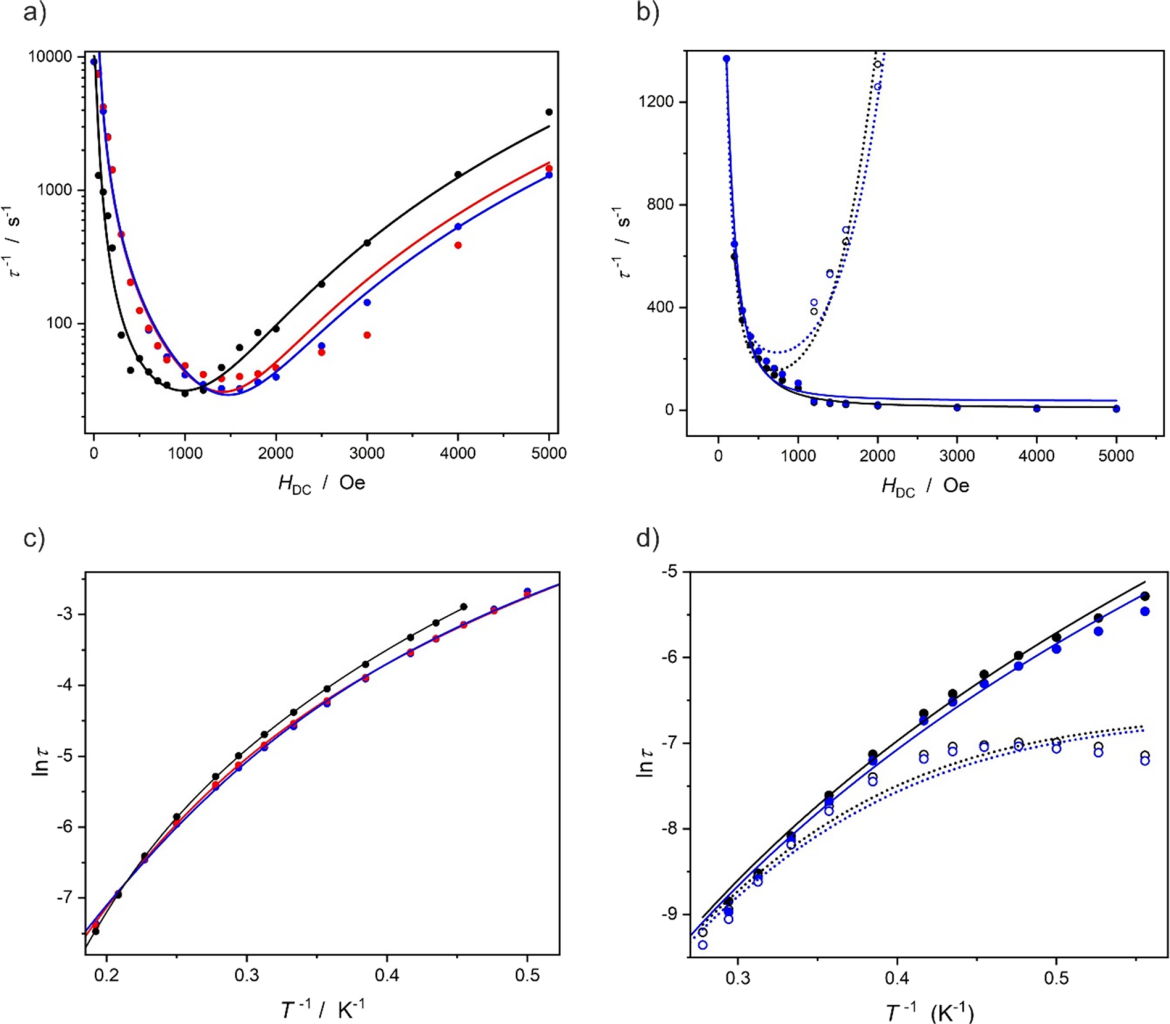
Magnetization relaxation times vs magnetic field in the form of τ^–1^(*H*) (a, b) and vs temperature in the form of ln τ(*T*^–1^) (c, d) for compound **1** before irradiation (black symbols and lines in (a) and (c)), after irradiation using violet light **1UV** (blue symbols and lines in (a) and (c)), and after irradiation using red light **1vis** (red symbols and lines in (a) and (c)). Panels (b) and (d) present the same dependencies for compound **2**: before irradiation (black symbols and lines in (b) and (d)) and after irradiation using violet light **2UV** (blue symbols and lines in (b) and (d)). The τ^–1^(*H*) data points were obtained by collecting AC magnetic susceptibility data at 2.5 K (a) and at 1.8 K (b), and the experimental ln τ(*T*^–1^) dependences were obtained at 800 Oe (c) and 500 Oe (full symbols and solid lines in (d)) or at 2000 Oe (empty symbols and dotted lines in (d)). The solid lines represent best fits using eq 1 for (a) and (b) or eq 2 for (c) and (d). Please note that in the case of (b), the relaxation splits into two branches which are fitted independently (for details, see the SI); in the case of (d), the temperature dependencies were recorded at two different magnetic fields and fitted simultaneously. The best fit parameters are gathered in their respective tables in the SI.

AC (alternating current) magnetic susceptibility measurements were performed at various external DC magnetic fields and temperatures as a function of the AC magnetic field frequency in the 1–1000 Hz range. Both compounds exhibit slow magnetic relaxation in the presence of a small external DC field typical for single-molecule magnets. In the case of **1**, the slow magnetization dynamics is slightly faster in zero DC magnetic field as compared to the parent compound [Er(BHT)_3_] reported by Yamashita et al.;^27^ however, the relaxation of the magnetization for **1** can be mildly altered by light irradiation due to the photochromic switching of the bridging dtepy ligand. All frequency dependencies were carefully analyzed by applying the Debye model. All details of the respective Debye fittings of the AC magnetic susceptibility data in the form of tables and figures can be found in the Supporting Information: **1** (Figure S3 and Tables S2 and S3), **1UV** (Figure S4 and Tables S4 and S5), **1vis** (Figure S5 and Tables S6 and S7), **2** (Figure S6 and Tables S8–S10), and **2UV** (Figure S7 and Tables S11–S13). For both compounds in different photoexcited/ photo-deexcited states, the respective magnetic field and temperature dependencies of the relaxation times were obtained and analyzed using the following equations (eqs 1 and 2), taking into account four different relaxation mechanisms possible for the Kramers ion Er^III^. The magnetic field dependence of the relaxation time was fitted using eq 1 and is presented in Figure 10a for **1** (black), **1UV** (blue), and **1vis** (red) as well as in Figure 10b for **2** (black) and **2UV** (blue):
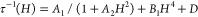
1where *A*_1_/(1 + *A*_2_*H*(2)) describes the quantum tunneling of magnetization (QTM), *B*_1_*H*^4^ describes direct relaxation, and *D* stands for processes that are magnetic field-independent.

The temperature dependence of the relaxation time was analyzed using eq 2 and is presented in Figure 10c for **1** (black), **1UV** (blue), and **1vis** (red) and in Figure 10d for **2** (black) and **2UV** (blue):
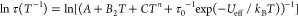
2where *A* stands for QTM, *B*_2_*T* describes the temperature dependence of the direct relaxation process and *B*_2_ = *B*_1_*H*^4^/*T*, *CT^n^* describes the Raman process, and τ_0_^–1^exp(−*U*_eff_/k_B_*T*))^−1^ is the Orbach relaxation process with τ_0_ as the attempt time of relaxation, *U*_eff_ as the effective energy barrier for the thermally activated magnetization reversal, *k*_B_ as the Boltzmann constant. The values of the best fit parameters to eqs 1 and 2 are gathered in separate Tables: Table S3 for **1**, Table S5 for **1UV**, Table S7 for **1vis**, Table S10 for **2**, and Table S13 for **2UV**.

Careful analysis of the τ^–1^(*H*) curves show important differences between the slow relaxation of the magnetization in **1** and **2**. The behavior of **1** is typical for isolated Er^III^ species with low coordination numbers, where the small applied magnetic field quenches the quantum tunneling of magnetization (QTM) and does not induce fast direct relaxation. Compound **2**, on the other hand, shows splitting of the χ″(ν) maxima above 1000 Oe, resulting in two τ^–1^(*H*) branches in Figure 10b. Such a behavior could be associated with the chain character of the compound. The influence of the UV light irradiation on the τ^–1^(*H*) curves is evident only in the case of compound **1**—the minimum of the τ^–1^(*H*) dependence after UV irradiation (sample **1UV**) moves toward slightly higher magnetic fields, which indicates that the QTM and direct relaxation processes are affected by the photocyclization of the dtepy ligands. However, the consecutive red light irradiation of **1UV**, which results in the opening of the dtepy as evidenced by the UV–vis and IR studies, does not result in the recovery of the original τ^–1^(*H*) shape in **1vis**. In other words, the slow relaxation of the magnetization in the pristine **1** and **1vis** are not identical from the point of view of τ^–1^(H) dependence, which means that the observed reversible photocyclization of the dtepy ligand is not transferred onto the magnetic properties.

The ln τ(*T*^–1^) dependencies indicate that the slow magnetization dynamics in both compounds are dominated by the Raman relaxation process—all curves presented in Figures 10c,d could be fitted without the contribution of the Orbach relaxation mechanism. The direct relaxation process operates only at very low temperatures. This is similar to the behavior of the parent compound [Er^III^(BHT)_3_(THF)].^26^ The analysis of the ln τ(*T*^–1^) dependencies before irradiation (**2**) and after 365 nm irradiation (**2UV**) suggest that the UV light has basically no effect on this chain system, in line with other results. This can be rationalized by the chain structure of **2**, which probably prevents the structural reorganization of the azopy ligand required by its photoisomerization. The behavior of **1**, on the other hand, deserves a more detailed discussion as the shape of the ln τ(*T*^–1^) for the pristine **1** is different from that of the UV-irradiated sample **1UV** (Figure 10c). The closer look at the ln τ(*T*^–1^) fitting parameters presented in Table S3 for **1** and Table S5 for **1UV** leads to the conclusion that the structural changes induced by light affect mainly the Raman exponent (4.92(4) for **1** and 4.3(3) for **1UV**). However, despite the observed reversibility of the photochromic behavior of **1** confirmed by UV–vis and IR spectroscopy, the AC magnetic susceptibility measurements reveal that red light irradiation does not allow the recovery of the initial SMM behavior. This is revealed by the shape of the τ^–1^(*H*) and ln τ(*T*^–1^) dependencies for **1vis** similar to **1UV** rather than to the pristine **1**. The possible explanation for this behavior is the collapse of the structural order in **1UV** during photocyclization of the dtepy (amorphization caused by UV light irradiation), which affects the AC magnetic properties.

To summarize, the AC magnetic susceptibility measurements for **1** before and after UV light irradiation reveal the change of the magnetization dynamics induced by light. This is a major advance as compared to the [Er^III^(BHT)_3_] parent compound reported by Yamashita et al.,^27^ which shows slightly slower relaxation of the magnetization but without the photo-switching functionality. It appears that the photocyclization of the ligand is mainly affecting the field dependence of the relaxation time at low temperature in compound **1**. This might be rationalized on the basis of slightly enhancing the electronic contact between the metal centers through the conjugated double bond system in the closed form of the dtepy molecule,^14,17^ which should mainly affect the QTM and direct relaxation processes as demonstrated for Ln-SMMs employing dtepy bridging ligand^16^ or other dte molecules.^15,40^ However, since the structure of the compound collapses after photoirradiation (the powder X-ray diffraction shows amorphization), it is necessary to design other more robust compounds that would recover the pristine structural order while the dte type ligands are being switched between the two forms.

## Conclusions

We have demonstrated that the rational assembly of low-coordinate lanthanide single-molecule magnets such as [Er^III^(BHT)_3_(THF)] with photochromic ligands like 1,2-bis(2-methyl-5-pyridyl)thien-3-yl)perfluorocyclopentene (dtepy) in a strictly non-coordinating hydrocarbon solvent is a rational way to photochromic nanomagnets where both desired functionalities—slow magnetic relaxation and photoswitching—are retained. Systems that combine dithienylethenes or azopyridine with lanthanides or transition metals^41^ are already present in the literature. Some of them^16,40^ perform better than compound **1** or **2**; however, the synthetic approach demonstrated herein is completely different compared to the literature reports as it relies on the use of low-coordinate lanthanide complexes with open coordination sites that can easily ″accept″ donor atoms from the photochromic ligands to form extended coordination systems via addition reactions and not substitution, which often complicates the self-assembly process leading to unpredictable geometries and serendipitous products.^40^

Moreover, compound **1** synthesized and investigated within this study demonstrates the influence of the photocyclization of the ligand on its nanomagnetic behavior. The observed photomagnetic effect is irreversible due to the loss of crystallinity of the compound but enables the formulation of a strategy toward new organometallic single-molecule magnets and molecular nanomagnets with high blocking temperatures^42^ that can be controlled by electromagnetic radiation. This approach will be further developed in our laboratories in order to achieve high-performance photoswitchable molecular magnets.

## Experimental Section

### General Considerations

All reactions and sample preparations were performed under high-purity Ar gas inside Inert PureLab HE glovebox. Solvents (HPLC) used in all syntheses were dried under Ar gas using Inert PureSolv EN7 solvent purification system and then stored over 3 Å molecular sieves for at least 24 h before use. [Er^III^(BHT)_3_(THF)]^26^ and [Er^III^(BHT)_3_]^27^ were prepared according to literature procedures. 1,2-bis(2-methyl-5-pyridyl)thien-3-yl)perfluorocyclopentene (dtepy) ligand was synthesized according to modified literature procedures^12,43,44^ starting from the commercially available substrates 2-methylthiophene (Aldrich) and 4-iodopyridine (TCI). Its preparation is described step-by-step in the Supporting Information, with ^1^H NMR spectra collected after each step (Figures S8–S10). 4,4′-Azopyridine (azopy) was obtained from commercial sources (Aldrich) and used as received.

### Preparation of {[Er^III^(BHT)_3_]_3_(Dtepy)_2_}^.^4C_5_H_12_ (**1**)

[Er^III^(BHT)_3_] (0.071 g, 0.087 mmol) was dissolved in *n*-pentane (18.0 g), and dtepy (0.030 g, 0.057 mmol) was added in one portion. The mixture was stirred for ca. 15 min until it becomes clear and was left for crystallization at room temperature. Small yellow crystals formed within 1–2 days. The identity and purity of the compound was confirmed by powder X-ray diffraction (PXRD) measurements (Figure 2). Yield: 30 mg (30%).

### Preparation of {[Er^III^(BHT)_3_(Azopy)}_n_·2C_5_H_12_ (**2**)

[Er^III^(BHT)_3_(THF)] (26.9 mg, 0.030 mmol) was dissolved in 3.8 g of pentane. 4,4′-Azopyridine (azopy) (5.5 mg, 0.030 mmol) was also dissolved in *n*-pentane (4.5 g). The orange solution of the ligand was added dropwise to the solution of the complex. The clear, dark-brown solution was sealed in a vial and left for crystallization at room temperature. The identity and purity of product was confirmed by PXRD measurements (Figure 2). Yield: 15 mg (43%).

### Single Crystal X-ray Diffraction

Single crystal X-ray diffraction data were collected using Oxford Diffraction SuperNova equipped with an Altas CCD detector for compound **1** at 130 and 270 K and Rigaku XtaLAB Synergy-S equipped with HyPix detector for compound **2** at 100 K. In both cases, Mo K_α_ radiation was used (PhotonJet microsources). Selected details of these measurements are presented in Table S1. Single crystals were transferred directly from the pentane solutions into the cryoprotectant oil and mounted on the goniometers using MiTeGen cryomounts. The measurements were performed twice for both compounds: first at low temperature (full data collection in Table S1) and then near room temperature at 270 K (full data collection in order to obtain structural model for comparison with the experimental PXRD patterns). ScXRD data were processed using CrysAlisPro 1.171.40.67a. The structures were solved using direct methods (intrinsic phasing using SHELXT^45^) and refined anisotropically using SHELX (weighted full-matrix least-squares on *F^2^*).^46^ Hydrogen atoms were placed in the calculated positions and refined as riding on the parent atoms. Mercury 2020.2.0 software (CCDC) was used to visualize the scXRD structural models and prepare the structural diagrams presented in the paper. CCDC 2175717 (compound **1** at 130 K), CCDC 2193239 (compound **1** at 270 K), and CCDC 2175718 (compound **2** at 100 K) contain the supplementary crystallographic data for this paper, which can be obtained free of charge from the Cambridge Crystallographic Data Centre via www.ccdc.cam.ac.uk/data_request/cif.

### Powder X-ray Diffraction

PXRD data were collected using a Bruker D8 Advance Eco diffractometer equipped with a Cu K_α_ radiation source and a Lynxeye silicon strip detector. The samples were ground using an agate mortar inside the glovebox and loaded into 0.7 or 0.5 mm glass capillaries under the mother solution. The capillaries were sealed using silicon and mounted on the goniometer head using beeswax. The PXRD of each sample was collected in at least three runs in the 3–50 2θ range to verify if the samples do not decompose during measurement. In both cases, no signs of decomposition were observed during the PXRD data collection. The experimental PXRD patterns are presented in Figure 2 and compared with the simulated patterns (CCDC Mercury 2020.2.0) from the structural models obtained using scXRD data collected at 270 K for **1** and 250 K for **2**.

### Magnetic Measurements

**Figure 11:**
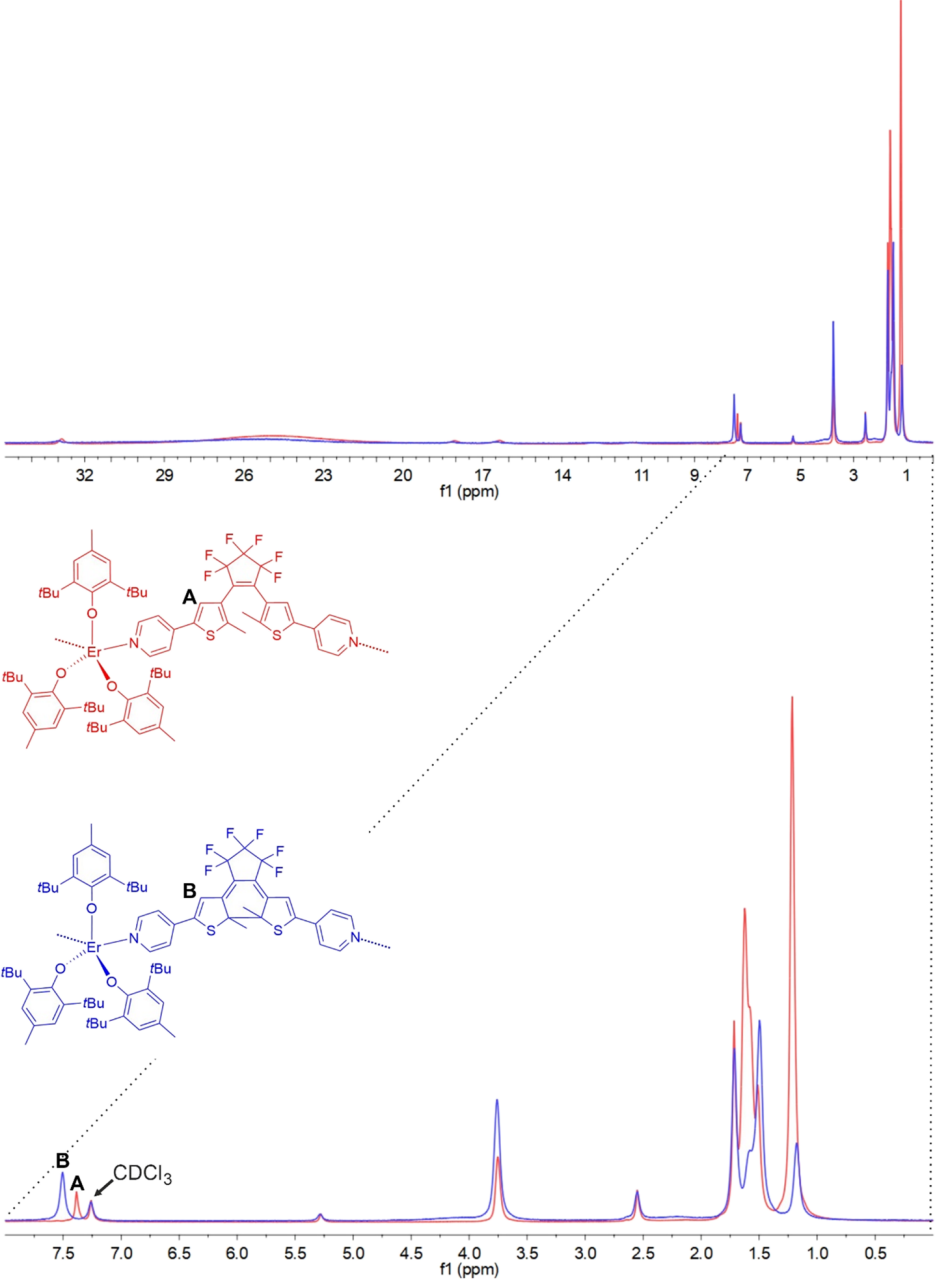
Comparison of the ^1^H NMR spectra in CDCl_3_ of compound **1** before irradiation (red line) and for **1UV** after 365 nm irradiation (blue line) with the indication of the thiophene proton shift related to the photocyclization of the dtepy ligand. Note that the irradiation of **1** using 365 nm light was performed on the bulk solid sample before it was dissolved in CDCl_3_ (for details, see the main text).

Magnetic data were recorded using a Quantum Design MPMS-3 Evercool magnetometer. The samples together with a quantity of pentane were loaded into borosilicate glass tubes (20–30 mg) in a setup that was described previously.^47^ The tubes with the samples were frozen in liquid nitrogen and then flame-sealed under vacuum to protect the compounds from the loss of crystallinity due to the rapid desolvation that could occur in the magnetometer sample chamber. The raw magnetic data were corrected for the diamagnetic contribution of the compounds^48^ and the solvent as well as the contribution of the glass tubes. Irradiation experiments were performed in the vacuum-sealed tubes at RT after removing them from the magnetometer sample chamber (ex situ). A 365 nm LED with 3 W power was used for the UV irradiations, and a 638 nm LED with 3 W power was used for visible light irradiations. The samples in the vacuum-sealed tubes were constantly rotated at ca. 2 RPM during the 15 h irradiation (overnight irradiation). Room-temperature cooling of the sample tubes was ensured by a small fan to avoid thermally activated side reactions. In order to assess the photoconversion **1** → **1UV** in the solid state, the blue **1UV** obtained after 15 h of 365 nm irradiation was isolated by filtering and dissolved in deuterated chloroform in the dark, and a ^1^H NMR experiment was performed. A similar experiment was performed for sample **1** before irradiation as a reference. The comparison of the two spectra after and before irradiation (Figure 11) shows some distinct changes. Some of the signals show significant paramagnetic shift (this applies to the pyridine arm protons and aromatic protons of the BHT ligands). The *tert*-butyl groups of the BHT ligands are only slightly affected by the photocyclization and overlap with the methyl groups of the dtepy ligand in the 1.5–3.5 ppm region, rendering it useless for the determination of the photoconversion. However, the signal associated with the thiophene proton designated A and B for the open and close form of the dtepy ligand, respectively (Figure 11), are well resolved and show that in the case of the spectra for the closed form after 15 h irradiation (blue line), there is no residual signal of the open form (red line). This directly proves that the photoconversion of the dtepy ligands in the bulk solid sample of compound **1** is close to 100%.

### UV–Vis and IR Spectroscopy

IR spectra were recorded using a Nicolet iN10 MX FTIR microscope for samples in the form of nujol mulls pressed between two 1.0 mm thick BaF_2_ plates. UV–vis experiments were done using a Shimadzu UV-3600i Plus spectrophotometer in the transmission mode for samples in the form of nujol mulls pressed between two 1.0 mm thick quartz plates. The irradiation experiments presented in Figures 6 and 7 were performed using the LEDs described in the ″Magnetic Measurements″ section without removing the samples from the instruments (in situ irradiation).

## Supporting Information Available

Additional tables with crystallographic parameters AC magnetic data fitting parameters; packing diagrams; AC magnetic susceptibility plots; ^1^H NMR spectra of the intermediate compounds for the synthesis of the dtepy ligand; dtepy preparation details (PDF)
