# Supplementary material for: Low-Coordinate Erbium(III) Single-Molecule Magnets with Photochromic Behavior
Source: Inorg Chem. 2022 Oct 5;61(41):16295–306. doi: 10.1021/acs.inorgchem.2c01999 (PMC9580000; doi:10.1021/acs.inorgchem.2c01999)
Supplement: Supplementary file 1 — ic2c01999_si_001.pdf [file ic2c01999_si_001.pdf]

Supporting Information

Low-Coordinate Erbium(III) Single-Molecule Magnets with Photochromic Behavior

Katarzyna Rogacz, Maria Brzozowska, Sebastian Baś, Katarzyna Kurpiewska, Dawid Pinkowicz*

Faculty of Chemistry, Jagiellonian University, Gronostajowa 2, 30-387 Kraków, Poland

* corresponding author's email address: dawid.pinkowicz@uj.edu.pl

Table S1. Selected crystallographic parameters for compounds **1** and **2**.

Compound	**1**	**1a**	**2**
Chemical formula	$\{[Er^{III}(BHT)_3]_3(dtepy)_2\}{\cdot}4C_5H_{12}$	$\{[Er^{III}(BHT)_3]_3(dtepy)_2\}{\cdot}4C_5H_{12}$	$\{[Er^{III}(BHT)_3(azopy){\cdot}2C_5H_{12}\}_n$
CCDC deposition number	2175717	2193239	2175718
Instrument	Oxford Diffraction SuperNova	Oxford Diffraction SuperNova	Rigaku XtaLAB Synergy S
Radiation	Mo Kα (λ = 0.71073 Å)	Mo Kα (λ = 0.71073 Å)	Mo Kα (λ = 0.71073 Å)
Formula	$C_{205}H_{287}Er_3F_{12}N_4O_9S_4$	$C_{205}H_{230}Er_3F_{12}N_4O_9S_4$	$C_{65}H_{101}ErN_4O_3$
M_r / g mol^{-1}	3809.39	3751.94	1153.75
T / K	130.0(1)	270.0(1)	100.00(13)
Crystal system	monoclinic	monoclinic	monoclinic
Space group	$P2_1/c$	$P2_1/c$	$P2_1/c$
a / Å	14.3062(3)	14.5311(2)	12.66390(10)
b / Å	24.9158(4)	25.2122(4)	27.17000(10)
c / Å	55.6257(8)	55.872(3)	18.38180(10)
α / °	90	90	90
β / °	95.5860(10)	95.556(2)	94.1770(10)
γ / °	90	90	90
V / Å^3	19733.6(6)	20373.0(12)	6307.98(7)
Z	4	4	4
ρ_{calc} / g cm^{-3}	1.282	1.223	1.215
μ / mm^{-1}	1.373	1.330	1.375
F (000)	7972	7744	2444
Crystal size / mm^3	0.12 × 0.06 × 0.04	0.12 × 0.06 × 0.04	0.12 × 0.04 × 0.03
ϑ range / °	2.545 – 28.753 (max)	2.547 – 25.027	2.158 - 26.865 (max)
Reflections collected	255 523	209 617	611 369
Independent reflections	47 624	35 921	13 567
R_{int}	0.1606	0.1693	0.0451
Parameters/restraints	2209/130	2199/199	842/180
R [$F_o > 2\sigma(F_o)$]	0.0770	0.0733	0.0487
wR (F^2)	0.1555	0.1997	0.1199
GOF on F^2	1.037	1.018	1.032
$\Delta\rho_{max}$, $\Delta\rho_{min}$ / e Å^{-3}	2.063/-1.200	1.928/-0.999	2.289/-3.580
Completeness / %	92.9	99.8	99.9

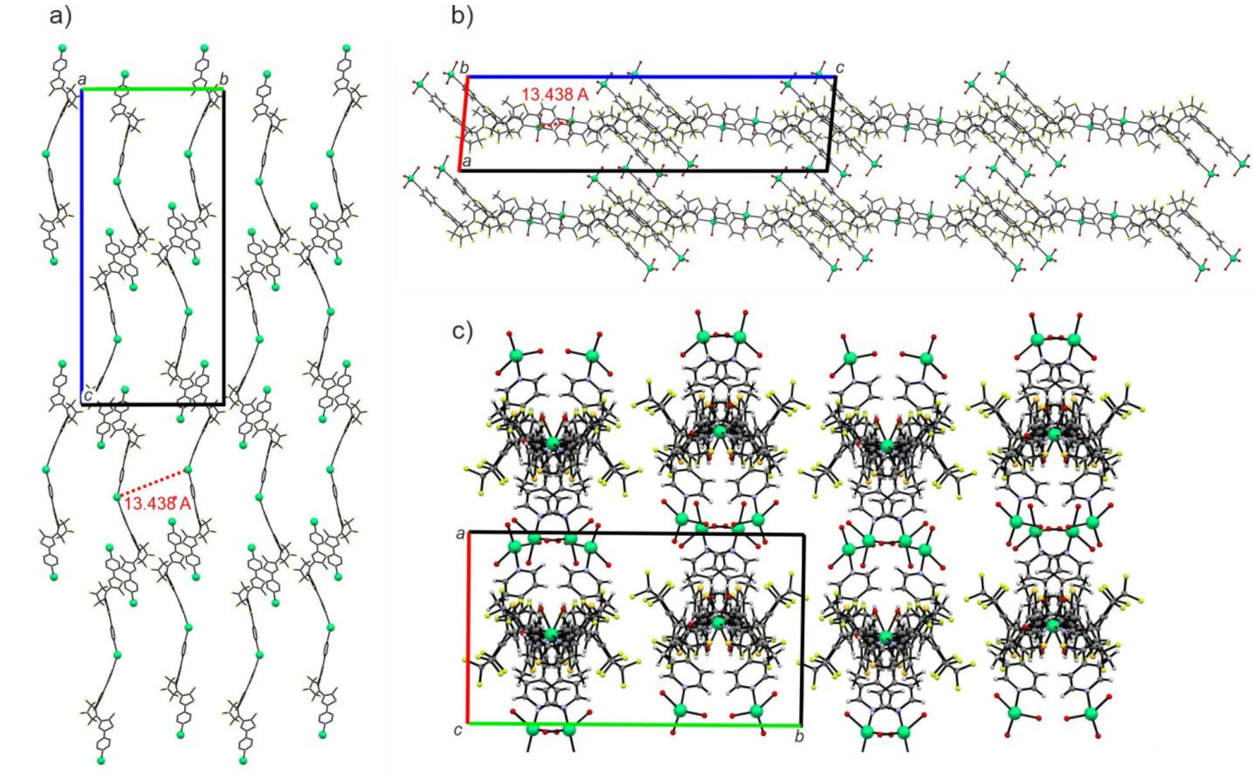

Figure S1. Packing diagram showing the crystal structure of **1** along the *a* (a), *b* (b) and *c* (c) crystallographic direction and the shortest intermolecular Er···Er distance of 13.437 Å. BHT ligands and H-atoms are omitted for clarity. Er – green balls.

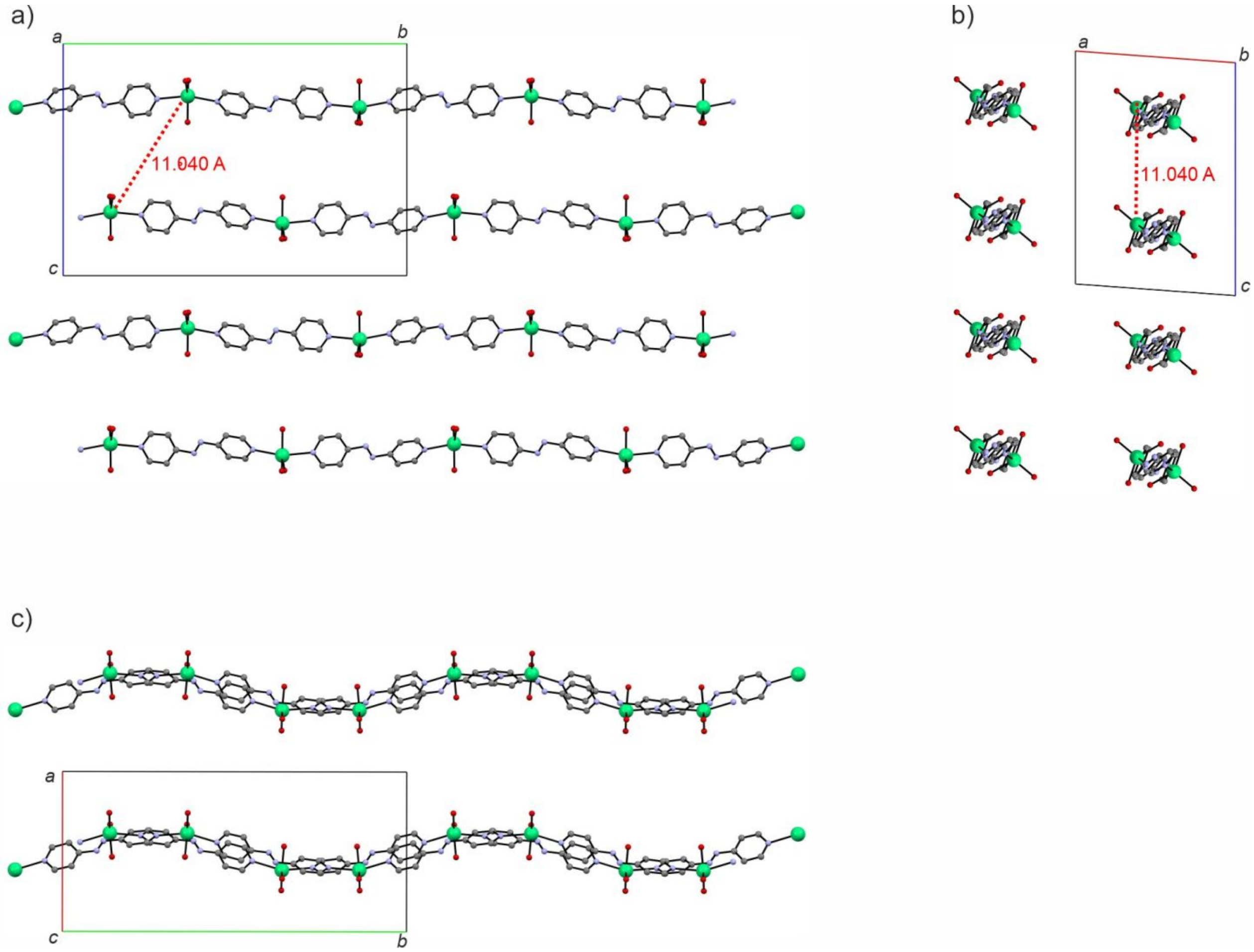

Figure S2. Packing diagram showing the crystal structure of **2** along the *a* (a), *b* (b) and *c* (c) crystallographic direction and the shortest intermolecular Er···Er distance of 11.040 Å. BHT ligands and H-atoms are omitted for clarity. Er – green balls, O – red balls, C – gray balls, N – light blue balls.

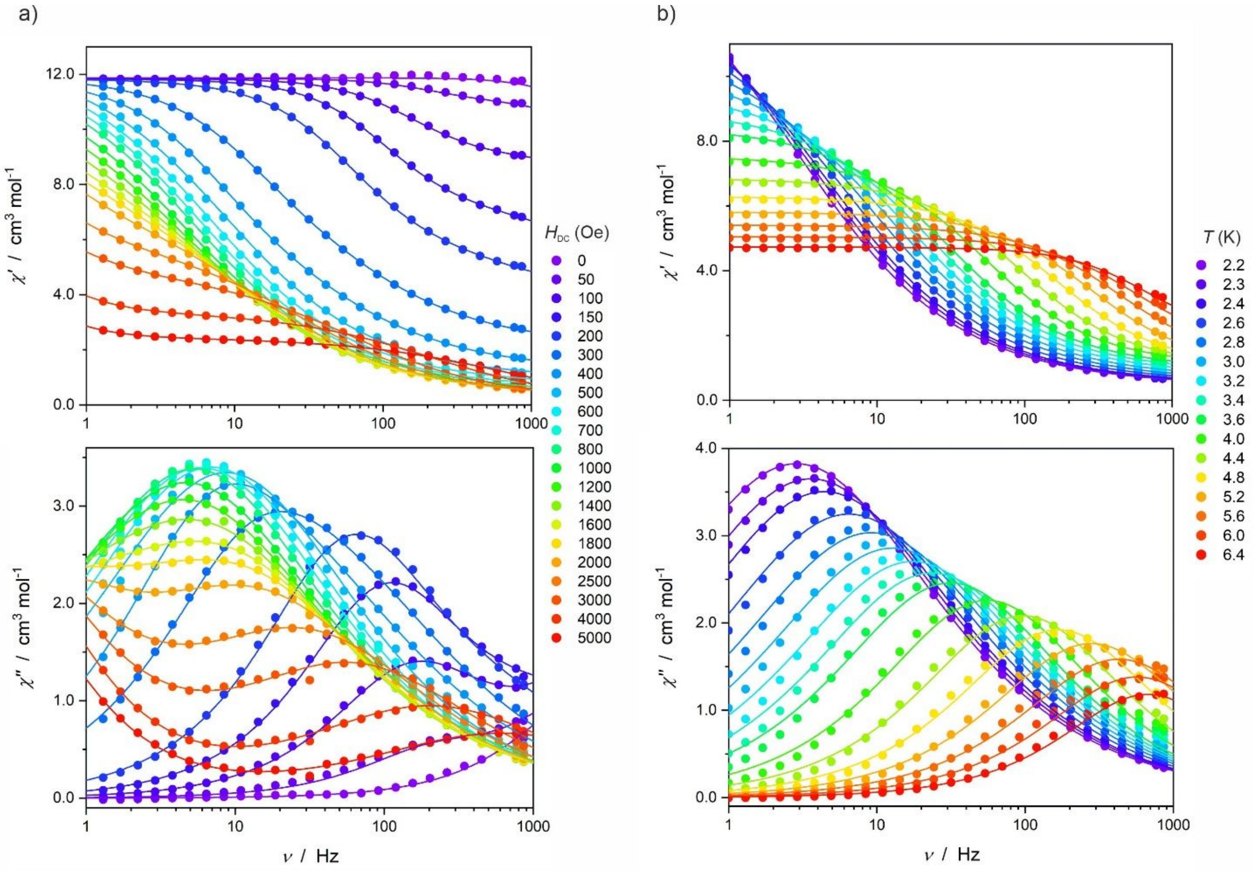

Figure S3. In-phase (χ') and out-of-phase (χ'') AC susceptibilities for **1** at 2.5 K measured in the 0-5000 Oe magnetic field range (a) and at 800 Oe in the 2.2-6.4 K temperature range (b). Values of α and τ parameters are gathered in Table S2. The solid lines are the best fits to generalized Debye model.

Table S2. Values of α and τ parameters obtained from generalized Debye model fitting of the $\tau(\nu)$ dependences for **1** at 2.5 K measured in the 0-5000 Oe magnetic field range depicted in Figure S3a (columns 1-5) and at 800 Oe in the 2.2-6.4 K temperature range depicted in Figure S3b (columns 6-10).

***T* = 2.5 K (Figure S3a)**					***H* = 800 Oe (Figure S3b)**				
***H* (Oe)**	*α*	*sd*	**τ (s)**	*sd*	***T* (K)**	*α*	*sd*	**τ (s)**	*sd*
**0**	0.00	0.0603	**$6.68\cdot10^{-5}$**	$2.63\cdot10^{-5}$	**2.2**	0.380	0.002	**$5.56\cdot10^{-2}$**	$5.1\cdot10^{-4}$
**50**	0.00	0.5236	**5.506E-5**	$8.02\cdot10^{-5}$	**2.3**	0.372	0.003	**$4.43\cdot10^{-2}$**	$4.9\cdot10^{-4}$
**100**	0.0894	0.4801	**1.032E-5**	$6.45\cdot10^{-5}$	**2.4**	0.364	0.003	**$3.60\cdot10^{-2}$**	$4.6\cdot10^{-4}$
**150**	0.2430	0.4461	**6.178E-5**	$6.79\cdot10^{-5}$	**2.6**	0.351	0.005	**$2.46\cdot10^{-2}$**	$4.2\cdot10^{-4}$
**200**	0.3151	0.2394	**2.326E-4**	$1.5\cdot10^{-4}$	**2.8**	0.338	0.007	**$1.74\cdot10^{-2}$**	$3.5\cdot10^{-4}$
**300**	0.3371	0.0274	**0.00258**	0.00119	**3.0**	0.321	0.008	**$1.25\cdot10^{-2}$**	$2.8\cdot10^{-4}$
**400**	0.3623	0.0210	**0.0045**	0.00177	**3.2**	0.303	0.009	**$9.1\cdot10^{-3}$**	$2.1\cdot10^{-4}$
**500**	0.342	0.007	**$1.82\cdot10^{-2}$**	$3.74\cdot10^{-4}$	**3.4**	0.28	0.01	**$6.8\cdot10^{-3}$**	$1.5\cdot10^{-4}$
**600**	0.345	0.006	**$2.30\cdot10^{-2}$**	$4.36\cdot10^{-4}$	**3.6**	0.261	0.009	**$5.1\cdot10^{-3}$**	$1.1\cdot10^{-4}$
**700**	0.352	0.005	**$2.69\cdot10^{-2}$**	$4.98\cdot10^{-4}$	**4.0**	0.226	0.008	**$2.87\cdot10^{-3}$**	$4.9\cdot10^{-5}$
**800**	0.31	0.04	**$2.89\cdot10^{-2}$**	$1.8\cdot10^{-3}$	**4.4**	0.205	0.008	**$1.65\cdot10^{-3}$**	$2.7\cdot10^{-5}$
**1000**	0.35	0.02	**$3.34\cdot10^{-2}$**	$1.5\cdot10^{-3}$	**4.8**	0.195	0.008	**$9.5\cdot10^{-4}$**	$1.8\cdot10^{-5}$
**1200**	0.36	0.08	**$3.15\cdot10^{-2}$**	$8.3\cdot10^{-3}$	**5.2**	0.190	0.009	**$5.7\cdot10^{-4}$**	$1.4\cdot10^{-5}$
**1400**	0.33	0.04	**$2.13\cdot10^{-2}$**	$3.7\cdot10^{-3}$	**5.6**	0.18	0.01	**$3.7\cdot10^{-4}$**	$1.2\cdot10^{-5}$
**1600**	0.31	0.02	**$1.51\cdot10^{-2}$**	$9.82\cdot10^{-4}$	**6.0**	0.15	0.01	**$2.8\cdot10^{-4}$**	$1.2\cdot10^{-5}$
**1800**	0.30	0.02	**$1.17\cdot10^{-2}$**	$5.56\cdot10^{-4}$	**6.4**	0.10	0.01	**$2.38\cdot10^{-4}$**	$9.5\cdot10^{-6}$
**2000**	0.36	0.004	**$1.09\cdot10^{-2}$**	$3.59\cdot10^{-4}$					
**2500**	0.32	0.003	**$5.06\cdot10^{-3}$**	$9.58\cdot10^{-5}$					
**3000**	0.32	0.01	$2.48\cdot10^{-3}$	$9.99\cdot10^{-5}$					
**4000**	0.33	0.01	$7.62\cdot10^{-4}$	$1.79\cdot10^{-4}$					
**5000**	0.36	0.02	$2.58\cdot10^{-4}$	$1.87\cdot10^{-4}$					

Table S3. Values of the best-fit parameters obtained by fitting the $\tau^{-1}(H)$ using Eq. 1 (top part of the table) and the $\tau(T)$ using Eq. 2 (bottom part of the table) for compound **1**. The relevant fits are shown as black lines in Figure 10a and 10c in the main text, respectively.

$\tau^{-1}(H) = A_1/(1+A_2H^2) + B_1H^4 + D$ (Eq. 1)

$\ln\tau(T^{-1}) = \ln[(A + B_2T + CT^n + \tau_0^{-1}\exp(-U_{eff}/k_BT))^{-1}]$ (Eq. 2)

Magnetic field dependence (black line in Figure 10a)	
***T* (K)**	2.5 K
**Frequency range (Hz)**	1-1000
**Field range (Oe)**	0-5000
**A_1 (s^{-1})**	10195(15399)
**A_2 (Oe^{-2})**	0.00116(183)
**B_1 (Oe^{-4})**	$4.8(2)\cdot10^{-12}$
**D (s^{-1})**	18(3)
**R^2**	0.948
Temperature dependence (black line in Figure 10c)	
**H_{DC} (Oe)**	800
**Frequency range (Hz)**	1-1000
**Temperature range (K)**	2.2-6.4
**A (s^{-1})**	0 (fixed)
**B_2 (s^{-1} K^{-1} Oe^{-1})**	0.788 (fixed)
**C (s^{-1} K^{-n})**	0.34(1)
n	4.92(4)
**R^2**	0.9999

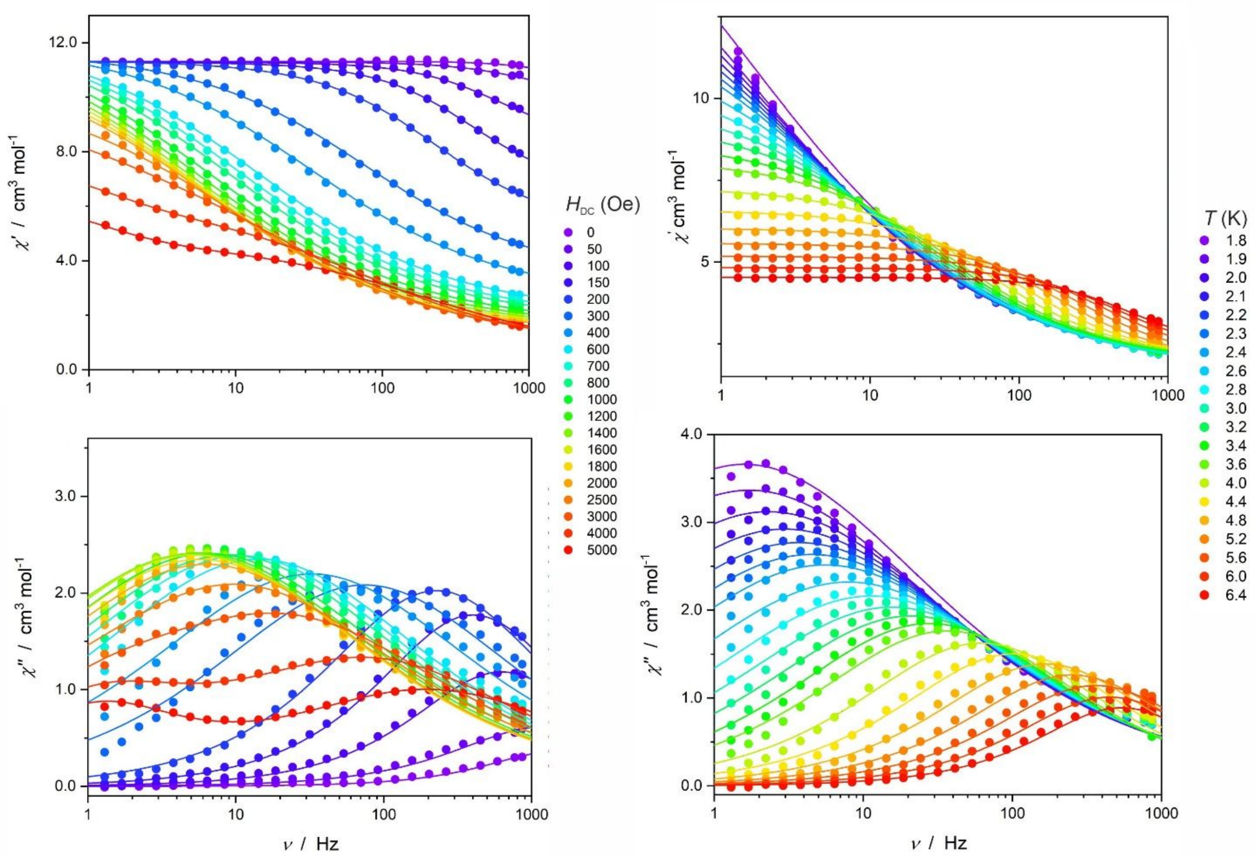

Figure S4. In-phase (χ') and out-of-phase (χ'') AC susceptibilities for **1UV** at 2.5 K measured in the 0-5000 Oe magnetic field range (a) and at 1000 Oe in the 1.8-6.4 K temperature range (b). Values of α and τ parameters are gathered in Table S3. The solid lines are the best fits to generalized Debye model.

Table S4. Values of α and τ parameters obtained from generalized Debye model fitting of the $\tau(\nu)$ dependences for **1UV** at 2.5 K measured in the 0-5000 Oe magnetic field range depicted in Figure S4a (columns 1-5) and at 1000 Oe in the 1.8-6.4 K temperature range depicted in Figure S4b (columns 6-10).

***T* = 2.5 K (Figure S4a)**					***H* = 1000 Oe (Figure S4b)**				
***H* (Oe)**	*α*	*sd*	***τ* (s)**	*sd*	***T* (K)**	*α*	*sd*	***τ* (s)**	*sd*
**0**	0	0.07	**$1.1\cdot10^{-4}$**	$3.3\cdot10^{-5}$	**1.8**	0.53	0.02	**0.10**	$2.3\cdot10^{-2}$
**50**	0.130	0.04	**$1.3\cdot10^{-4}$**	$2.2\cdot10^{-5}$	**1.9**	0.53	0.01	**$9.2\cdot10^{-2}$**	$7.6\cdot10^{-3}$
**100**	0.133	0.01	**$2.5\cdot10^{-4}$**	$1.0\cdot10^{-5}$	**2.0**	0.52	0.01	**$6.9\cdot10^{-2}$**	$4.8\cdot10^{-3}$
**150**	0.175	0.007	**$4.0\cdot10^{-4}$**	$7.2\cdot10^{-6}$	**2.1**	0.52	0.01	**$5.4\cdot10^{-2}$**	$3.4\cdot10^{-3}$
**200**	0.258	0.007	**$7.0\cdot10^{-4}$**	$1.3\cdot10^{-5}$	**2.2**	0.51	0.01	**$4.3\cdot10^{-2}$**	$2.3\cdot10^{-3}$
**300**	0.411	0.009	**$2.14\cdot10^{-3}$**	$6.4\cdot10^{-5}$	**2.3**	0.50	0.01	**$3.5\cdot10^{-2}$**	$1.7\cdot10^{-3}$
**400**	0.45	0.01	**$4.9\cdot10^{-3}$**	$1.6\cdot10^{-4}$	**2.4**	0.49	0.01	**$2.9\cdot10^{-2}$**	$1.3\cdot10^{-3}$
**600**	0.458	0.009	**$1.12\cdot10^{-2}$**	$3.9\cdot10^{-4}$	**2.6**	0.48	0.01	**$2.01\cdot10^{-2}$**	$8.3\cdot10^{-4}$
**700**	0.470	0.009	**$1.47\cdot10^{-2}$**	$5.4\cdot10^{-3}$	**2.8**	0.45	0.01	**$1.41\cdot10^{-2}$**	$5.4\cdot10^{-4}$
**800**	0.474	0.009	**$1.78\cdot10^{-2}$**	$7.1\cdot10^{-4}$	**3.0**	0.43	0.01	**$1.02\cdot10^{-2}$**	$3.6\cdot10^{-4}$
**1000**	0.485	0.008	**$2.4\cdot10^{-2}$**	$1.1\cdot10^{-3}$	**3.2**	0.40	0.01	**$7.6\cdot10^{-3}$**	$2.5\cdot10^{-4}$
**1200**	0.486	0.009	**0.02868**	$1.4\cdot10^{-3}$	**3.4**	0.37	0.01	**$5.8\cdot10^{-3}$**	$1.7\cdot10^{-4}$
**1400**	0.485	0.008	**0.03073**	$1.5\cdot10^{-3}$	**3.6**	0.35	0.01	**$4.4\cdot10^{-3}$**	$1.2\cdot10^{-4}$
**1600**	0.481	0.008	**0.03075**	$1.4\cdot10^{-3}$	**4.0**	0.30	0.01	**$2.59\cdot10^{-3}$**	$6.4\cdot10^{-5}$
**1800**	0.472	0.008	**0.02753**	$1.2\cdot10^{-3}$	**4.4**	0.27	0.01	**$1.57\cdot10^{-3}$**	$3.9\cdot10^{-5}$
**2000**	0.472	0.007	**0.02511**	$9.5\cdot10^{-4}$	**4.8**	0.25	0.01	**$9.6\cdot10^{-4}$**	$2.7\cdot10^{-5}$
**2500**	0.43	0.35	**$1.47\cdot10^{-2}$**	$1.4\cdot10^{-2}$	**5.2**	0.22	0.01	**$6.3\cdot10^{-4}$**	$2.0\cdot10^{-5}$
**3000**	0.45	0.01	$6.94\cdot10^{-3}$	$1.2\cdot10^{-3}$	**5.6**	0.20	0.01	**$4.4\cdot10^{-4}$**	$1.6\cdot10^{-5}$
**4000**	0.41	0.01	$1.87\cdot10^{-3}$	$6.5\cdot10^{-5}$	**6.0**	0.15	0.01	**$3.5\cdot10^{-4}$**	$1.3\cdot10^{-5}$
**5000**	0.41	0.01	$7.65\cdot10^{-4}$	$2.2\cdot10^{-4}$	**6.4**	0.10	0.01	**$2.96\cdot10^{-4}$**	$9.5\cdot10^{-6}$

Table S5. Values of the best-fit parameters obtained by fitting the $\tau^{-1}(H)$ using Eq. 1 (top part of the table) and the $\tau(T)$ using Eq. 2 (bottom part of the table) for compound **1UV**. The relevant fits are shown as blue lines in Figure 10a and 10c in the main text, respectively.

$$\tau^{-1}(H) = A_1/(1+A_2H^2) + B_1H^4 + D \qquad \text{(Eq. 1)}$$

$$\ln\tau(T^{-1}) = \ln[(A + B_2T + CT^n + \tau_0^{-1}\exp(-U_{eff}/k_BT))^{-1}] \qquad \text{(Eq. 2)}$$

Magnetic field dependence<br>(blue line in Figure 10a)	
***T* (K)**	2.5 K
**Frequency range (Hz)**	1-1000
**Field range (Oe)**	0-5000
**A_1 (s^{-1})**	26320(17185)
**A_2 (Oe^{-2})**	$6(4)\cdot10^{-4}$
**B_1 (Oe^{-4})**	$2.1(3)\cdot10^{-12}$
***D* (s^{-1})**	0 (fixed)
**R^2**	0.905
Temperature dependence<br>(blue line in Figure 10c)	
**H_{DC} (Oe)**	1000
**Frequency range (Hz)**	1-1000
**Temperature range (K)**	1.8-6.4
***A* (s^{-1})**	0 (fixed)
**B_2 (s^{-1} K^{-1} Oe^{-1})**	0.8206 (fixed)
***C* (s^{-1} K^{-n})**	0.68(16)
n	4.3(3)
**R^2**	0.99882

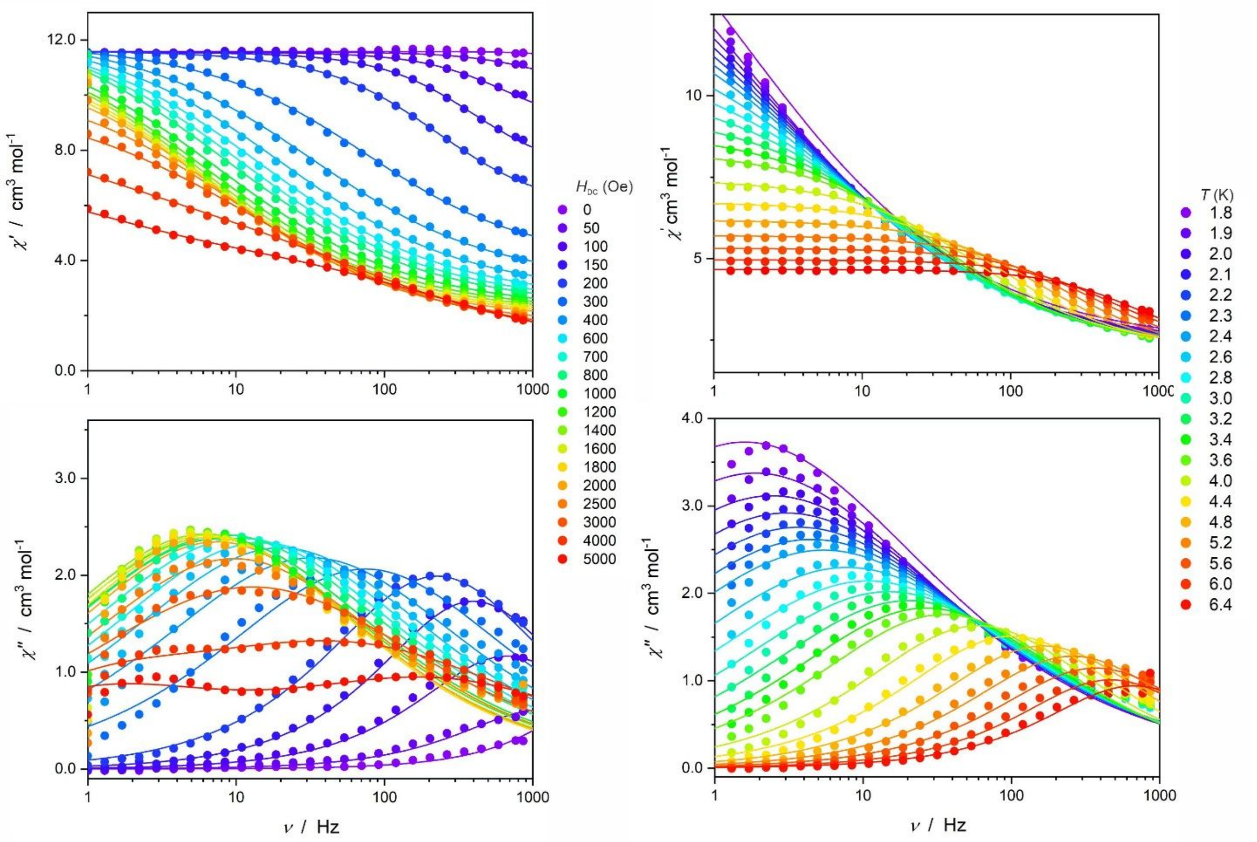

Figure S5. In-phase (*χ*') and out-of-phase (*χ*'') AC susceptibilities for **1vis** at 2.5 K measured in the 0-5000 Oe magnetic field range (a) and at 1000 Oe in the 1.8-6.4 K temperature range (b). Values of α and τ parameters are gathered in Table S6. The solid lines are the best fits to generalized Debye model.

Table S6. Values of α and τ parameters obtained from generalized Debye model fitting of the $\tau(\nu)$ dependences for **1vis** at 2.5 K measured in the 0-5000 Oe magnetic field range depicted in Figure S5a (columns 1-5) and at 1000 Oe in the 1.8-6.4 K temperature range depicted in Figure S5b (columns 6-10).

***T* = 2.5 K (Figure S5a)**					***H* = 1000 Oe (Figure S5b)**				
***H* (Oe)**	*α*	*sd*	**τ (s)**	*sd*	***T* (K)**	*α*	*sd*	**τ (s)**	*sd*
**50**	0.09	0.04	$\mathbf{1.3{\cdot}10^{-4}}$	$2.2{\cdot}10^{-5}$	**1.8**	0.52	0.02	**0.1**	$2.5{\cdot}10^{-3}$
**100**	0.13	0.02	$\mathbf{2.4{\cdot}10^{-4}}$	$1.1{\cdot}10^{-5}$	**1.9**	0.52	0.01	$\mathbf{8.5{\cdot}10^{-2}}$	$8.1{\cdot}10^{-3}$
**150**	0.165	0.008	$\mathbf{4.0{\cdot}10^{-4}}$	$8.7{\cdot}10^{-6}$	**2.0**	0.51	0.01	$\mathbf{6.6{\cdot}10^{-2}}$	$5.3{\cdot}10^{-3}$
**200**	0.248	0.007	$\mathbf{7.0{\cdot}10^{-4}}$	$1.4{\cdot}10^{-5}$	**2.1**	0.51	0.01	$\mathbf{5.2{\cdot}10^{-2}}$	$3.7{\cdot}10^{-3}$
**300**	0.39	0.01	$\mathbf{2.14{\cdot}10^{-3}}$	$7.4{\cdot}10^{-5}$	**2.2**	0.50	0.01	$\mathbf{4.3{\cdot}10^{-2}}$	$2.7{\cdot}10^{-5}$
**400**	0.43	0.01	$\mathbf{4.9{\cdot}10^{-3}}$	$1.6{\cdot}10^{-4}$	**2.3**	0.50	0.01	$\mathbf{3.6{\cdot}10^{-2}}$	$2.1{\cdot}10^{-3}$
**600**	0.44	0.01	$\mathbf{8.0{\cdot}10^{-3}}$	$2.7{\cdot}10^{-4}$	**2.4**	0.49	0.01	$\mathbf{2.9{\cdot}10^{-2}}$	$1.5{\cdot}10^{-3}$
**700**	0.44	0.01	$\mathbf{1.08{\cdot}10^{-2}}$	$4.3{\cdot}10^{-4}$	**2.6**	0.47	0.01	$\mathbf{2.05{\cdot}10^{-2}}$	$9.5{\cdot}10^{-4}$
**800**	0.45	0.01	$\mathbf{1.46{\cdot}10^{-2}}$	$7.0{\cdot}10^{-4}$	**2.8**	0.45	0.01	$\mathbf{1.47{\cdot}10^{-2}}$	$6.1{\cdot}10^{-4}$
**1000**	0.46	0.01	$\mathbf{1.9{\cdot}10^{-2}}$	$1.1{\cdot}10^{-3}$	**3.0**	0.43	0.01	$\mathbf{1.07{\cdot}10^{-2}}$	$4.0{\cdot}10^{-4}$
**1200**	0.45	0.02	$\mathbf{2.1{\cdot}10^{-2}}$	$1.4{\cdot}10^{-3}$	**3.2**	0.40	0.01	$\mathbf{7.9{\cdot}10^{-3}}$	$2.7{\cdot}10^{-4}$
**1400**	0.45	0.02	$\mathbf{2.4{\cdot}10^{-2}}$	$1.8{\cdot}10^{-3}$	**3.4**	0.37	0.01	$\mathbf{6.0{\cdot}10^{-3}}$	$1.8{\cdot}10^{-4}$
**1600**	0.434	0.02	$\mathbf{2.6{\cdot}10^{-2}}$	$2.6{\cdot}10^{-3}$	**3.6**	0.34	0.01	$\mathbf{4.5{\cdot}10^{-3}}$	$1.3{\cdot}10^{-4}$
**1800**	0.43	0.02	$\mathbf{2.5{\cdot}10^{-2}}$	$2.6{\cdot}10^{-3}$	**4.0**	0.29	0.01	$\mathbf{2.66{\cdot}10^{-3}}$	$6.3{\cdot}10^{-5}$
**2000**	0.42	0.03	$\mathbf{2.4{\cdot}10^{-2}}$	$2.5{\cdot}10^{-3}$	**4.4**	0.26	0.01	$\mathbf{1.58{\cdot}10^{-5}}$	$3.7{\cdot}10^{-5}$
**2500**	0.43	1.02	$\mathbf{1.64{\cdot}10^{-2}}$	$4.37{\cdot}10^{-2}$	**4.8**	0.23	0.01	$\mathbf{9.7{\cdot}10^{-4}}$	$2.4{\cdot}10^{-5}$
**3000**	0.49	0.39	$\mathbf{1.22{\cdot}10^{-2}}$	$1.96{\cdot}10^{-2}$	**5.2**	0.22	0.01	$\mathbf{6.2{\cdot}10^{-4}}$	$1.8{\cdot}10^{-5}$
**4000**	0.50	0.05	$\mathbf{2.58{\cdot}10^{-3}}$	$1.14{\cdot}10^{-3}$	**5.6**	0.20	0.01	$\mathbf{4.3{\cdot}10^{-4}}$	$1.7{\cdot}10^{-5}$
**5000**	0.46	0.08	$\mathbf{6.86{\cdot}10^{-4}}$	$1.69{\cdot}10^{-4}$	**6.0**	0.15	0.01	$\mathbf{3.4{\cdot}10^{-4}}$	$1.4{\cdot}10^{-5}$
					**6.4**	0.14	0.02	$\mathbf{2.4{\cdot}10^{-4}}$	$1.6{\cdot}10^{-5}$

Table S7. Values of the best-fit parameters obtained by fitting the $\tau^{-1}(H)$ using Eq. 1 (top part of the table) and the $\tau(T)$ using Eq. 2 (bottom part of the table) for compound **1vis**. The relevant fits are shown as red lines in Figure 10a and 10c in the main text, respectively.

$$\tau^{-1}(H) = A_1/(1+A_2H^2) + B_1H^4 + D \qquad \text{(Eq. 1)}$$

$$\ln\tau(T^{-1}) = \ln[(A + B_2T + CT^n + \tau_0^{-1}\exp(-U_{eff}/k_BT))^{-1}] \qquad \text{(Eq. 2)}$$

Magnetic field dependence (red line in Figure 10a)	
***T* (K)**	2.5 K
**Frequency range (Hz)**	1-1000
**Field range (Oe)**	0-5000
$\mathbf{A_1}$ **(s^{-1})**	63288
$\mathbf{A_2}$ **(Oe^{-2})**	0.0016(10)
$\mathbf{B_1}$ **(Oe^{-4})**	$2.6(12){\cdot}10^{-12}$
D **(s^{-1})**	$1.14{\cdot}10^{-13}$ (fixed)
$\mathbf{R^2}$	0.878
Temperature dependence (red line in Figure 10c)	
$\mathbf{H_{DC}}$ **(Oe)**	1000
**Frequency range (Hz)**	1-1000
**Temperature range (K)**	1.8-6.4
A **(s^{-1})**	0 (fixed)
$\mathbf{B_2}$ **(s^{-1} K^{-1} Oe^{-1})**	1.03 (fixed)
C **(s^{-1} K^{-n})**	0.60(8)
n	4.49(15)
$\mathbf{R^2}$	0.999

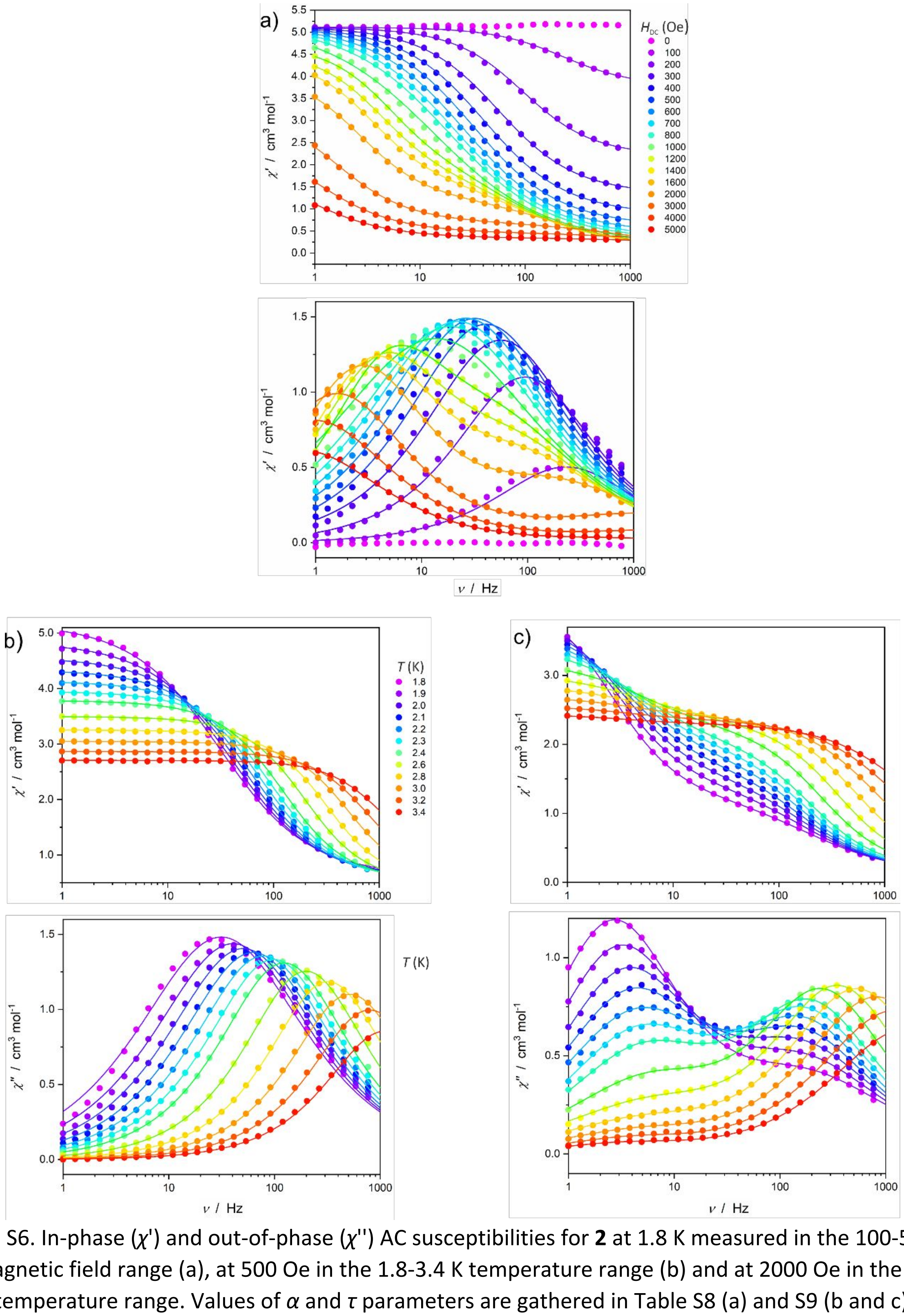

Figure S6. In-phase (χ') and out-of-phase (χ'') AC susceptibilities for **2** at 1.8 K measured in the 100-5000 Oe magnetic field range (a), at 500 Oe in the 1.8-3.4 K temperature range (b) and at 2000 Oe in the 1.8-3.6 K temperature range. Values of α and τ parameters are gathered in Table S8 (a) and S9 (b and c). The solid lines are the best fits to generalized Debye model.

Table S8. Values of α and τ parameters obtained from generalized Debye model fitting of the $\tau(\nu)$ dependences for **2** at 1.8 K measured in the 100-5000 Oe magnetic field range depicted in Figure S6a.

***T* = 1.8 K (Figure S6a)**								
***H* (Oe)**	*α*	*sd*	***τ* (s)**	*sd*				
**100**	0.1825	0.0216	**0.00069**	0.00009				
**200**	0.1802	0.0066	**0.00167**	0.00003				
**300**	0.2206	0.0068	**0.00284**	0.00005				
**400**	0.2466	0.0069	**0.00392**	0.00007				
**500**	0.2691	0.0061	**0.00500**	0.00007				
**600**	0.2869	0.0060	**0.00610**	0.00009				
**700**	0.3077	0.0063	**0.00728**	0.00011				
**800**	0.3291	0.0069	**0.00856**	0.00016				
**1000**	0.3770	0.0083	**0.01173**	0.00030				
	α	*sd*	***τ* (s)**	*sd*	α_2	*sd*	**τ_2 (s)**	*sd*
**1200**	0.1273	0.0084	**0.03223**	0.00059	0.291	0.010	**0.00256**	0.00014
**1400**	0.1554	0.0053	**0.03772**	0.00038	0.270	0.008	**0.00186**	0.00006
**1600**	0.1358	0.0088	**0.04542**	0.00060	0.287	0.015	**0.00156**	0.00007
**2000**	0.1567	0.0084	**0.05935**	0.00065	0.298	0.020	**0.000772**	0.000026
**3000**	0.1883	0.0103	**0.09998**	0.00262	0.383	0.051	**0.000115**	0.000043
**4000**	0.2302	0.0046	**0.16189**	0.00342	0.369	0.038	**0.000061**	0.000028
**5000**	0.2488	0.0071	**0.20793**	0.00982	0.163	0.079	**0.000237**	0.000065

Table S9. Values of α and τ parameters obtained from generalized Debye model fitting of the $\tau(\nu)$ dependences for **2** at 500 Oe in the 1.8-3.4 K temperature range depicted in Figure S6b (columns 1-5) and at 2000 Oe in the 1.8-3.6 K temperature range depicted in Figure S6c (columns 6-10).

***H* = 500 Oe (Figure S6b)**					***H* = 2000 Oe (Figure S6c)**				
***T* (K)**	*α*	*sd*	***τ* (s)**	*sd*	***T* (K)**	*α*	*sd*	***τ* (s)**	*sd*
**1.8**	0.00507	0.000072	**0.2739**	0.0062	**1.8**	0.1722	0.0059	**0.06126**	0.00052
**1.9**	0.00393	0.000043	**0.2469**	0.0050	**1.9**	0.1805	0.0056	**0.05254**	0.00046
**2.0**	0.00314	0.000028	**0.2223**	0.0042	**2.0**	0.1852	0.0050	**0.04587**	0.00039
**2.1**	0.00254	0.000019	**0.2002**	0.0036	**2.1**	0.2030	0.0058	**0.04127**	0.00044
**2.2**	0.00203	0.000014	**0.1784**	0.0034	**2.2**	0.2263	0.0066	**0.03766**	0.00050
**2.3**	0.00162	0.000010	**0.1584**	0.0031	**2.3**	0.2045	0.0090	**0.03608**	0.00065
**2.4**	0.00129	0.000007	**0.1406**	0.0029	**2.4**	0.2191	0.0086	**0.03354**	0.00060
**2.6**	0.0008017	0.0000046	**0.1119**	0.0029	**2.6**	0.2584	0.0088	**0.03104**	0.00060
**2.8**	0.0004968	0.0000032	**0.0932**	0.0029	**2.8**	0.3059	0.0155	**0.0301**	0.0011
**3.0**	0.0003085	0.0000030	**0.0886**	0.0035	**3.0**	0.2883	0.0155	**0.0338**	0.0011
**3.2**	0.0002003	0.0000034	**0.0880**	0.0048	**3.2**	0.2605	0.0235	**0.0379**	0.0016
**3.4**	0.0001443	0.0000045	**0.0838**	0.0069	**3.4**	0.1944	0.0393	**0.0432**	0.0027
					**3.6**	0.1644	0.0504	**0.0488**	0.0038

Table S10. Values of the best-fit parameters obtained by fitting the $\tau^{-1}(H)$ using Eq. 1 (top part of the table) and the $\tau(T)$ using Eq. 2 (bottom part of the table) for compound **2**. The relevant fits are shown as black lines in Figure 10b and 10d in the main text, respectively.

$\tau^{-1}(H) = A_1/(1+A_2H^2) + B_1H^4 + D$ (Eq. 1)

$\ln\tau(T^{-1}) = \ln[(A + B_2T + CT^n + \tau_0^{-1}\exp(-U_{eff}/k_BT))^{-1}]$ (Eq. 2)

Magnetic field dependence (black lines in Figure 10b)		
	branch 1	branch 2
T **(K)**	1.8 K	1.8 K
**Frequency range (Hz)**	1-1000	1-1000
**Field range (Oe)**	200 – 5000	200 – 2000
A_1 **(s^{-1})**	967(98)	6585(51871)
A_2 **(Oe^{-2})**	1.7(4)·10^{-5}	0.0003(26)
B_1 **(Oe^{-4})**	0 (fixed)	7.9(6)·10^{-11}
D **(s^{-1})**	10(10)	107(45)
R^2	0.9876	0.9685
**Temperature dependence (black lines in Figure 10d)**		
H_{DC} **(Oe)**	500	2000
**Frequency range (Hz)**	1 – 1000	1 – 1000
**Temperature range (K)**	1 – 3.4	1 – 3.6
A **(s^{-1})**	0 (fixed)	733(65)
B_2 **(s^{-1}K^{-1}Oe^{-1})**	0 (fixed)	
C **(s^{-1}K^{-n})**	6(1)	
n	5.7(2)	
R^2	0.9794	

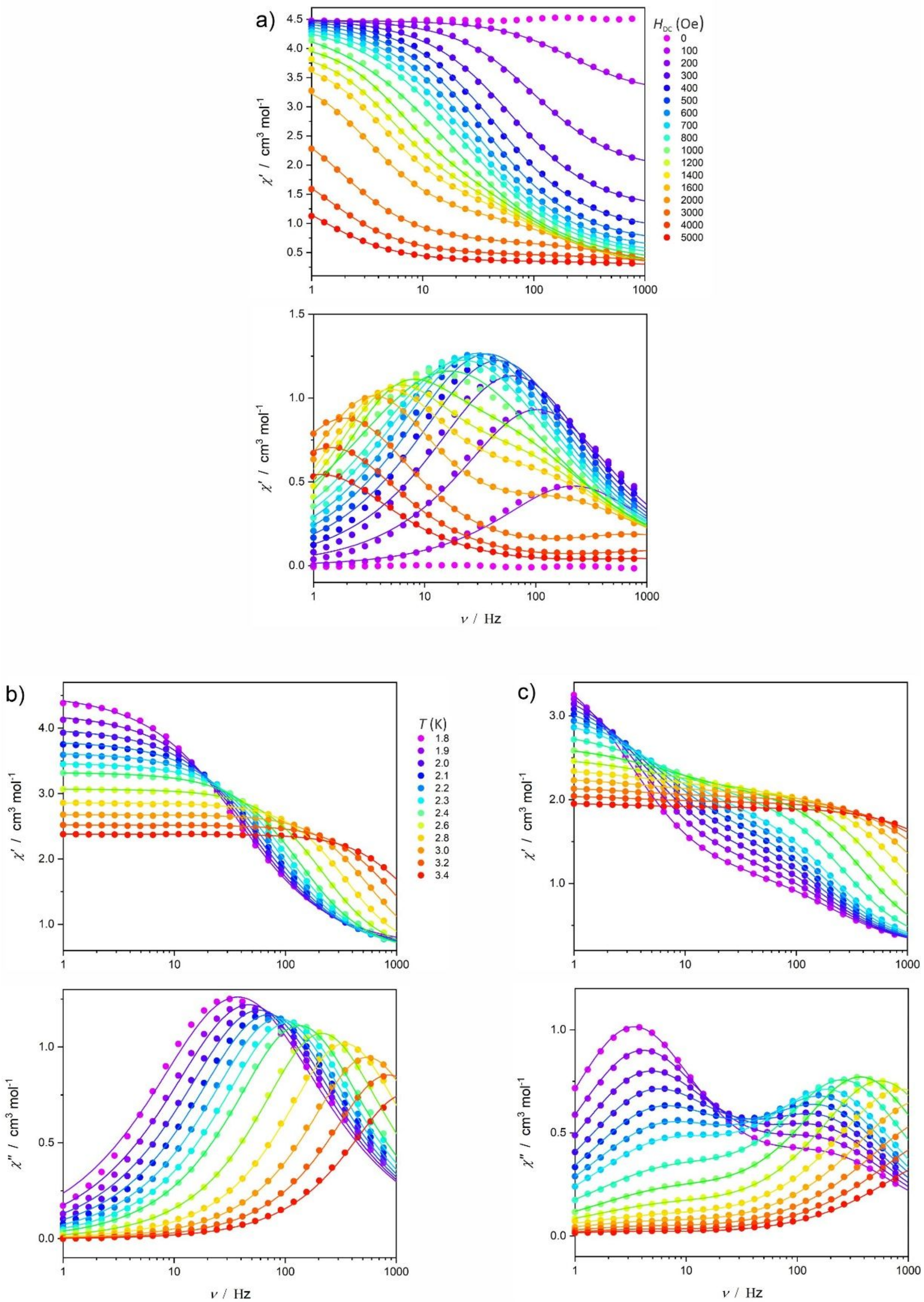

Figure S7. In-phase (χ') and out-of-phase (χ'') AC susceptibilities for **2UV** at 1.8 K measured in the 100-5000 Oe magnetic field range (a), at 500 Oe in the 1.8-3.4 K temperature range (b) and at 2000 Oe in the 1.8-3.6 K temperature range. Values of α and τ parameters are gathered in Table S11 (a) and S12 (b and c). The solid lines are the best fits to generalized Debye model.

Table S11. Values of α and τ parameters obtained from generalized Debye model fitting of the $\tau(\nu)$ dependences for **2UV** at 1.8 K measured in the 100-5000 Oe magnetic field range depicted in Figure S7a.

***T* = 1.8 K (Figure S7a)**								
***H* (Oe)**	α	*sd*	**τ (s)**	*sd*				
**100**	0.1807	0.0104	**0.00073**	0.00002				
**200**	0.2139	0.0077	**0.00154**	0.00003				
**300**	0.2373	0.0076	**0.00257**	0.00004				
**400**	0.2559	0.0074	**0.00348**	0.00006				
**500**	0.2697	0.0068	**0.00434**	0.00007				
**600**	0.2851	0.0067	**0.00522**	0.00008				
**700**	0.3025	0.0069	**0.00613**	0.00010				
**800**	0.3223	0.0074	**0.00711**	0.00013				
**1000**	0.3637	0.0092	**0.00944**	0.00024				
	α	*sd*	**τ (s)**	*sd*	α_2	*sd*	**τ_2 (s)**	*sd*
**1200**	0.1207	0.0136	**0.02677**	0.00082	0.295	0.018	**0.00238**	0.00023
**1400**	0.1258	0.0109	**0.03184**	0.00070	0.286	0.018	**0.00189**	0.00013
**1600**	0.1355	0.0101	**0.03693**	0.00065	0.272	0.021	**0.00142**	0.00008
**2000**	0.1425	0.0091	**0.04789**	0.00059	0.257	0.027	**0.000794**	0.000035
**3000**	0.1816	0.0032	**0.08364**	0.00050	0.248	0.024	**0.000185**	0.000016
**4000**	0.1769	0.0047	**0.11228**	0.00120	0.289	0.070	**0.000087**	0.000040
**5000**	0.1844	0.0064	**0.13644**	0.00259	0.165	0.130	**0.000145**	0.000075

Table S12. Values of α and τ parameters obtained from generalized Debye model fitting of the $\tau(\nu)$ dependences for **2UV** at 500 Oe in the 1.8-3.4 K temperature range depicted in Figure S7b (columns 1-5) and at 2000 Oe in the 1.8-4.0 K temperature range depicted in Figure S7c (columns 6-10), respectively.

***H* = 500 Oe (Figure S7b)**					***H* = 2000 Oe (Figure S7c)**				
***T* (K)**	α	*sd*	**τ (s)**	*sd*	***T* (K)**	α	*sd*	**τ (s)**	*sd*
**1.8**	0.00425	6.5E-05	**0.2691**	0.0067	**1.8**	0.1767	0.0023	**0.04884**	0.00017
**1.9**	0.00337	4.3E-05	**0.2445**	0.0058	**1.9**	0.1909	0.0038	**0.04189**	0.00028
**2.0**	0.00274	2.9E-05	**0.2216**	0.0050	**2.0**	0.2054	0.0027	**0.03682**	0.00019
**2.1**	0.00224	2.2E-05	**0.2001**	0.0046	**2.1**	0.2075	0.0035	**0.03342**	0.00025
**2.2**	0.00183	1.5E-05	**0.1780**	0.0040	**2.2**	0.2147	0.0063	**0.03100**	0.00043
**2.3**	0.00148	1.1E-05	**0.1587**	0.0038	**2.3**	0.2299	0.0054	**0.02890**	0.00036
**2.4**	0.00119	7.7E-06	**0.1391**	0.0033	**2.4**	0.2415	0.0059	**0.02753**	0.00040
**2.6**	7.428E-04	4.7E-06	**0.1073**	0.0031	**2.6**	0.2811	0.0108	**0.02631**	0.00071
**2.8**	4.632E-04	3.1E-06	**0.0839**	0.0030	**2.8**	0.2788	0.0158	**0.02590**	0.00095
**3.0**	2.909E-04	2.9E-06	**0.0725**	0.0037	**3.0**	0.2952	0.0179	**0.02797**	0.00110
**3.2**	1.875E-04	3.4E-06	**0.0723**	0.0050	**3.2**	0.3131	0.0295	**0.03237**	0.00200
**3.4**	1.273E-04	4.4E-06	**0.0715**	0.0072	**3.4**	0.2398	0.0438	**0.04095**	0.00309
					**3.6**	0.2054	0.0572	**0.04529**	0.00424
					**3.8**	0.1348	0.0805	**0.04748**	0.00575
					**4.0**	0.1632	0.1090	**0.05718**	0.01014

Table S13. Values of the best-fit parameters obtained by fitting the $\tau^{-1}(H)$ using Eq. 1 (top part of the table) and the $\tau(T)$ using Eq. 2 (bottom part of the table) for compound **2UV**. The relevant fits are shown as blue lines in Figure 10b and 10d in the main text, respectively.

$$\tau^{-1}(H) = A_1/(1+A_2H^2) + B_1H^4 + D \qquad \text{(Eq. 1)}$$

$$\ln\tau(T^{-1}) = \ln[(A + B_2T + CT^n + \tau_0^{-1}\exp(-U_{eff}/k_BT))^{-1}] \qquad \text{(Eq. 2)}$$

Magnetic field dependence (blue lines in Figure 10b)		
	branch 1	branch 2
**T (K)**	1.8 K	1.8 K
**Frequency range (Hz)**	1-1000	1-1000
**Field range (Oe)**	100 – 5000	100 – 2000
**A_1 (s^{-1})**	1966(134)	2439(682)
**A_2 (Oe^{-2})**	$4.8(8)\cdot10^{-5}$	$9.7(53)\cdot10^{-5}$
**B_1 (Oe^{-4})**	0	$7.4(5)\cdot10^{-11}$
**D (s^{-1})**	36(14)	134(38)
**R^2**	0.9897	0.9730
Temperature dependence (blue lines in Figure 10d)		
**H_{DC} (Oe)**	500	2000
**Frequency range (Hz)**	1 – 1000	1 – 1000
**Temperature range (K)**	1 – 3.6	1 – 4.0
**A (s^{-1})**	0 (fixed)	750(78)
**B_2 ($s^{-1}K^{-1}Oe^{-1}$)**	0 (fixed)	
**C ($s^{-1}K^{-n}$)**	7.4(14)	
**n**	5.5(2)	
**R^2**	0.9731	

Step-by-step preparation of 1,2-bis(2-methyl-5-pyridyl)thien-3-yl)perfluorocyclopentene (dtepy) according to a modified literature procedure[1]

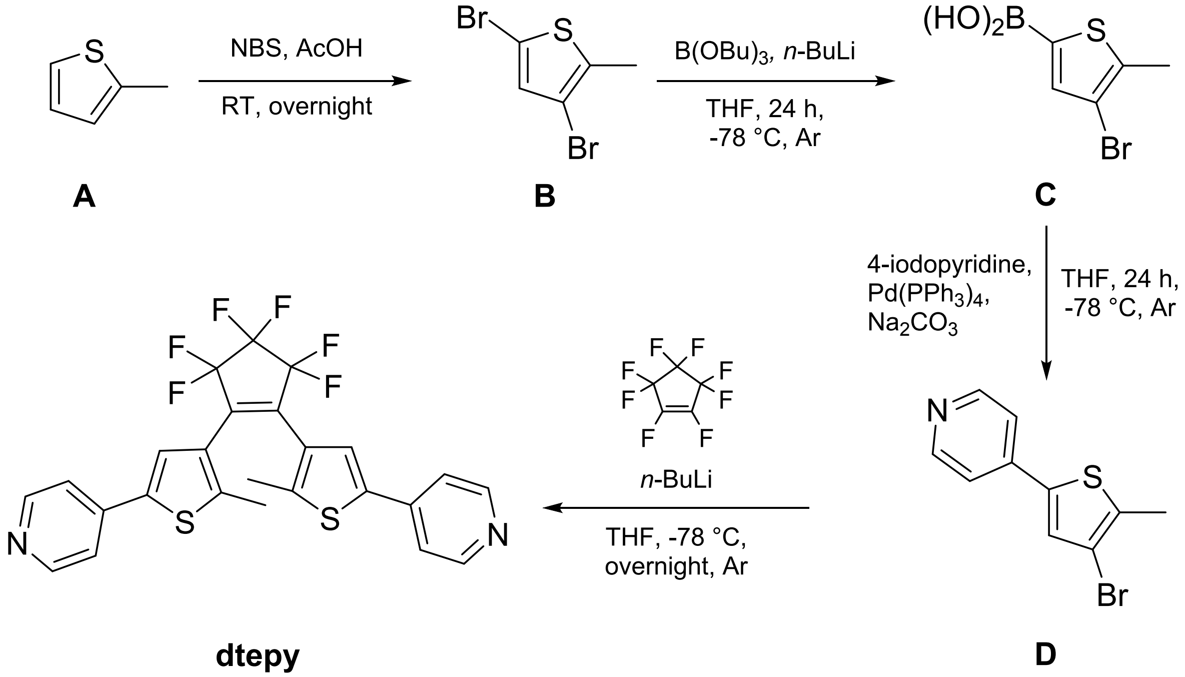

Scheme 1. Step-by-step preparation of 1,2-bis(2-methyl-5-pyridyl)thien-3-yl)perfluorocyclopentene (dtepy) starting from the commercially available 2-methylthiophene (**A**). Selected abbreviations: **B** = 3,5-dibromo-2-methylthiophene, **C** = (4-bromo-5-methylthiophen2-yl)boronic acid, **D** = 4-(4-bromo-5-methylthiophen-2-yl)pyridine, NBS = N-bromosuccinimide (Merck), $B(OBu)_3$ = tributylborate (Merck), *n*-BuLi = 2.5 M n-butyllithium in hexanes (Merck).

Preparation of 3,5-dibromo-2-methylthiophene (B) according to the modified literature procedure[2]

N-bromosuccinimide (9.05 g, 50.85 mmol, 2 eq) was dissolved in 60 ml of glacial acetic acid. Then the solution of 2-methylthiophene (2.46 ml, 25.42 mmol, 1 eq) in 25 ml of glacial acetic acid was added dropwise while stirring. The reaction mixture was stirred overnight at room temperature and then the content of the flask was poured into 400 ml of hexane and water mixture (1:1 by volume). The organic phase was separated and washed with 1 M NaOH and brine. The organic phase was dried over $MgSO_4$ and evaporated using rotavap. The obtained yellow oil was purified by vacuum distillation (90-110°C, 10 mbar) yielding 4.94 g of a colorless oil (76%). ^1H NMR (600 MHz, $CDCl_3$, Figure S8) δ 6.86 (s, 1H), 2.34 (s, 3H).

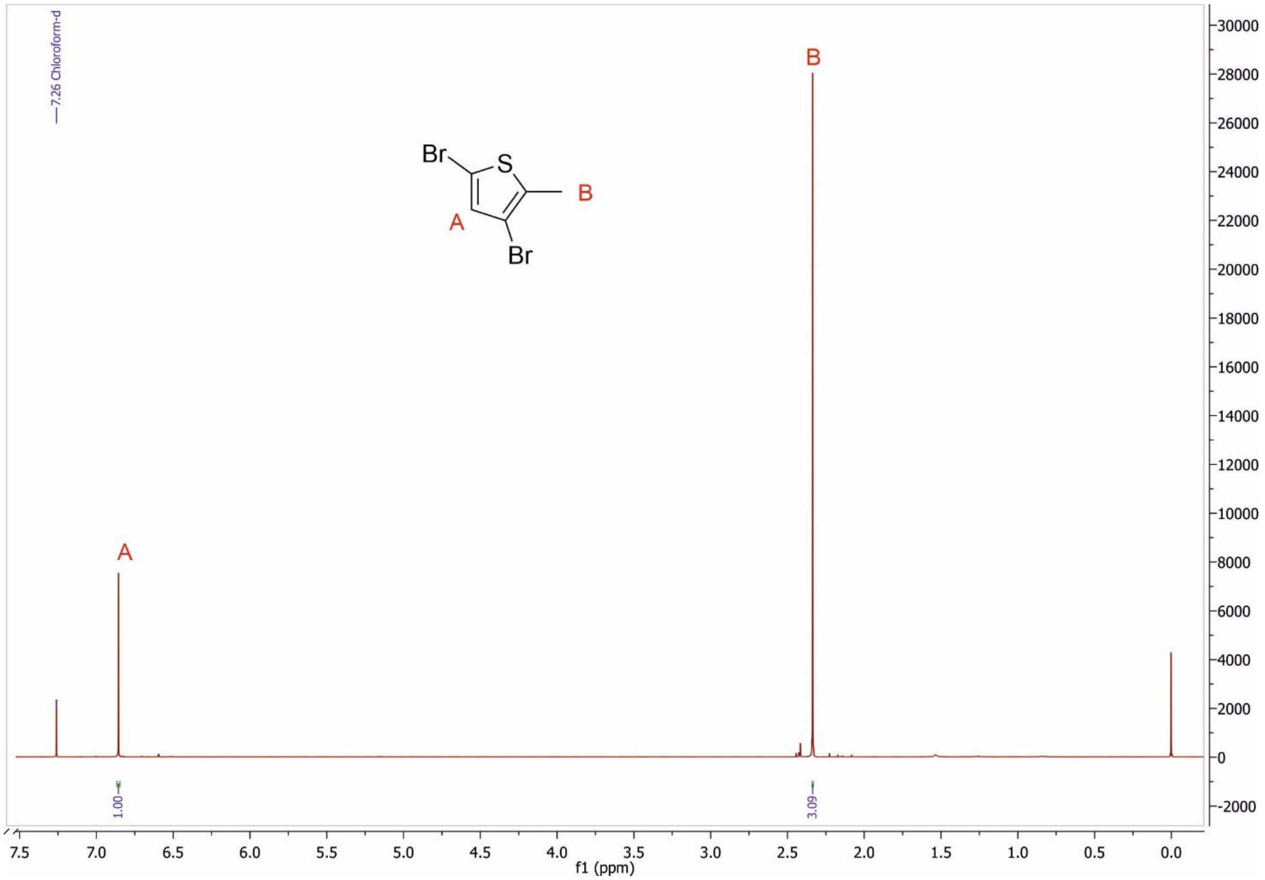

Figure S8. ^1H NMR spectrum of 3,5-dibromo-2-methylthiophene (**B**) recorded in $CDCl_3$ with TMS as the reference.

Preparation of (4-bromo-5-methylthiophen2-yl)boronic acid (C)

The following reaction was performed under inert gas atmosphere. 3,5-dibromo-2-methylthiophene (**B**) (9.7 g, 37.9 mmol, 1 eq) was dissolved in anhydrous THF in a two-necked round bottom flask. The mixture was cooled to -78 °C. To the stirred solution 2.5 M *n*-BuLi (16 ml, 39.8 mmol, 1.5 eq) was added dropwise. The reaction was stirred for 15 minutes at -78 °C and then tributyl borate (10.46 g, 45.5 mmol, 1.2 eq) was slowly added to the mixture followed by slow warm up to RT and further stirring at RT for 24h. The reaction mixture was quenched with 110 ml of 1M HCl and stirred for additional 1 h. Then THF was removed under vacuum and the mixture was extracted with Et_2O (3 × 50 ml). The organic layers were combined and the boronic acid was removed by washing with 2M NaOH solution (3 × 50 ml). The combined aqueous layers were carefully acidified to neutral using conc. HCl. The obtained off-white-to-yellow solid was filtered and dried under vacuum (yield: 5.5 g, 76%) and used without purification for the preparation of 4-(4-bromo-5-methylthiophen-2-yl)pyridine (**D**) as described in the next step described below. Crude ^{1}H NMR (300 MHz, DMSO; Figure S9) δ 8.28 (s, 2H), 7.50 (s, 1H), 2.37 (s, 3H).

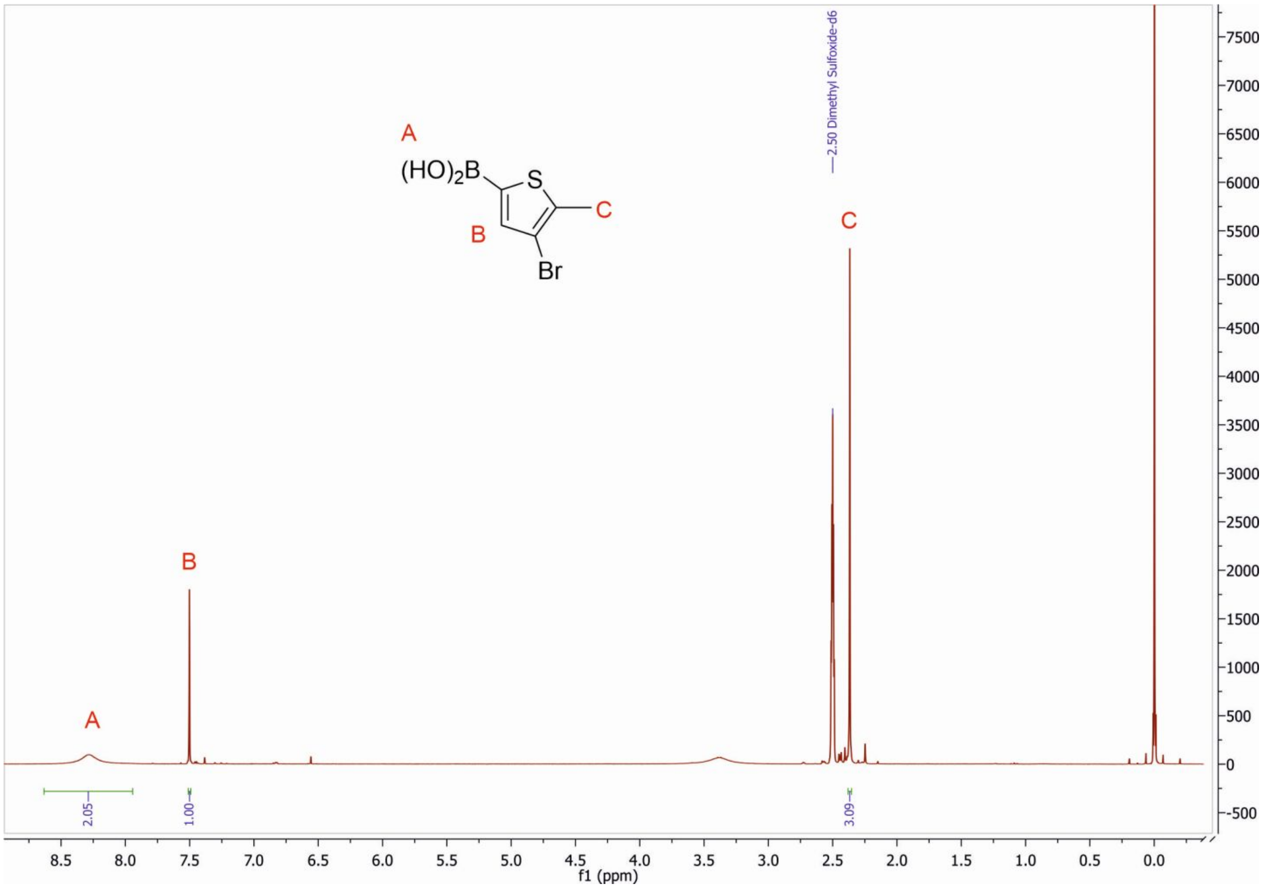

Figure S9. ^{1}H NMR spectrum of (4-bromo-5-methylthiophen2-yl)boronic acid (**C**) recorded in $CDCl_3$ with TMS as the reference.

Preparation of 4-(4-bromo-5-methylthiophen-2-yl)pyridine (D)

The (4-bromo-5-methylthiophen2-yl)boronic acid (**C**) (4.0 g, 18.1 mmol, 1 eq), 4-iodopyridine (4.45 g, 21.7 mmol, 1.2 eq) and 50 ml THF were placed in a 200 ml Schlenk flask udner inert atmosphere. Into the stirred solution under argon 50 ml of 20% Na_2CO_2 solution in degassed water was added and the reaction mixture was degassed again by performing three vacuum-inert gas cycles. $Pd(PPh_3)_4$ (1.04 g, 0.9 mmol, 5 mol%) was added and the reaction mixture was heated to 85 °C for 72 h under inert gas. After cooling to RT, the product was extracted from the mixture by two 50 ml portions of dichloromethane and the organic layers were washed with H_2O (2 × 50 ml) and brine (50 ml). After drying over $MgSO_4$ the solvent was removed under vacuum yielding an orange solid as the crude product. Purification by silica gel column chromatography (EtOAc) afforded a light yellow solid (4.32 g, 93% yield) as the final product. ^1H NMR (300 MHz, $CDCl_3$; Figure S10) δ 8.58 (d, *J* = 5.8 Hz, 2H), 7.36 (dd, *J* = 4.6, 1.6 Hz, 2H), 7.30 (s, 1H), 2.44 (s, 3H).

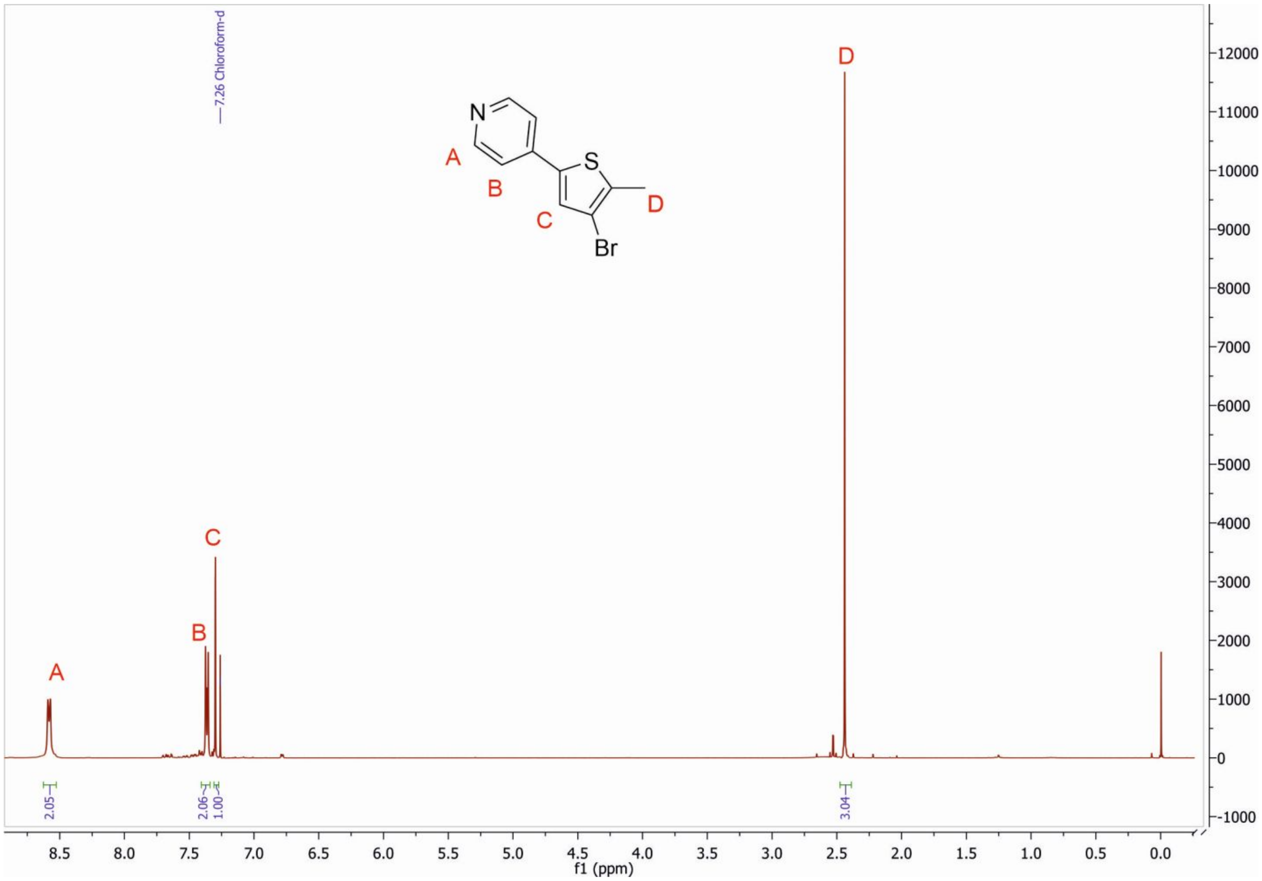

Figure S10. ^1H NMR spectrum of 4-(4-bromo-5-methylthiophen-2-yl)pyridine (**D**) recorded in $CDCl_3$ with TMS as the reference

Preparation of 1,2-bis(2'-methyl)-5'-(pyrid-4''-yl)thien-3'-yl)perfluorocyclopentene (dtepy)

4-(4-bromo-5-methylthiophen-2-yl)pyridine (**D**) (3.60 g, 14.2 mmol, 2 eq) was dissolved in 150 ml of anhydrous THF and 2.5 *n*-BuLi in hexanes (5.66 ml, 14.2 mmol, 2eq) was slowly added at -78 °C udner inert gas atmosphere. The solution turns deep red and perfluorocyclopentene (neat, 1.50 g, 7.1 mmol, 1 eq) was then slowly added using a cooled syringe. The green mixture is allowed to warm to RT and then stirred overnight. After that 40 ml of 1 M HCl was added and the reaction stirred for additional 30 min. THF was removed under vacuum and the residue was neutralized using saturated aqueous Na_2CO_3 followed by a few extractions using DCM (3 × 50 ml) and additional washings with saturated Na_2CO_3 (50 ml) and brine (50 ml). The product was purified by column chromatography over alumina (DCM/AcOEt = 4:1) yielding a greenish solid which becomes white after washing with pentane (0.50 g, 14%). ^{1}H NMR (300 MHz, $CDCl_3$, Figure S11) δ 8.62 (dd, *J* = 4.6, 1.5 Hz, 4H), 7.48 (s, 2H), 7.43 (dd, *J* = 4.6, 1.6 Hz, 4H), 2.01 (s, 6H).

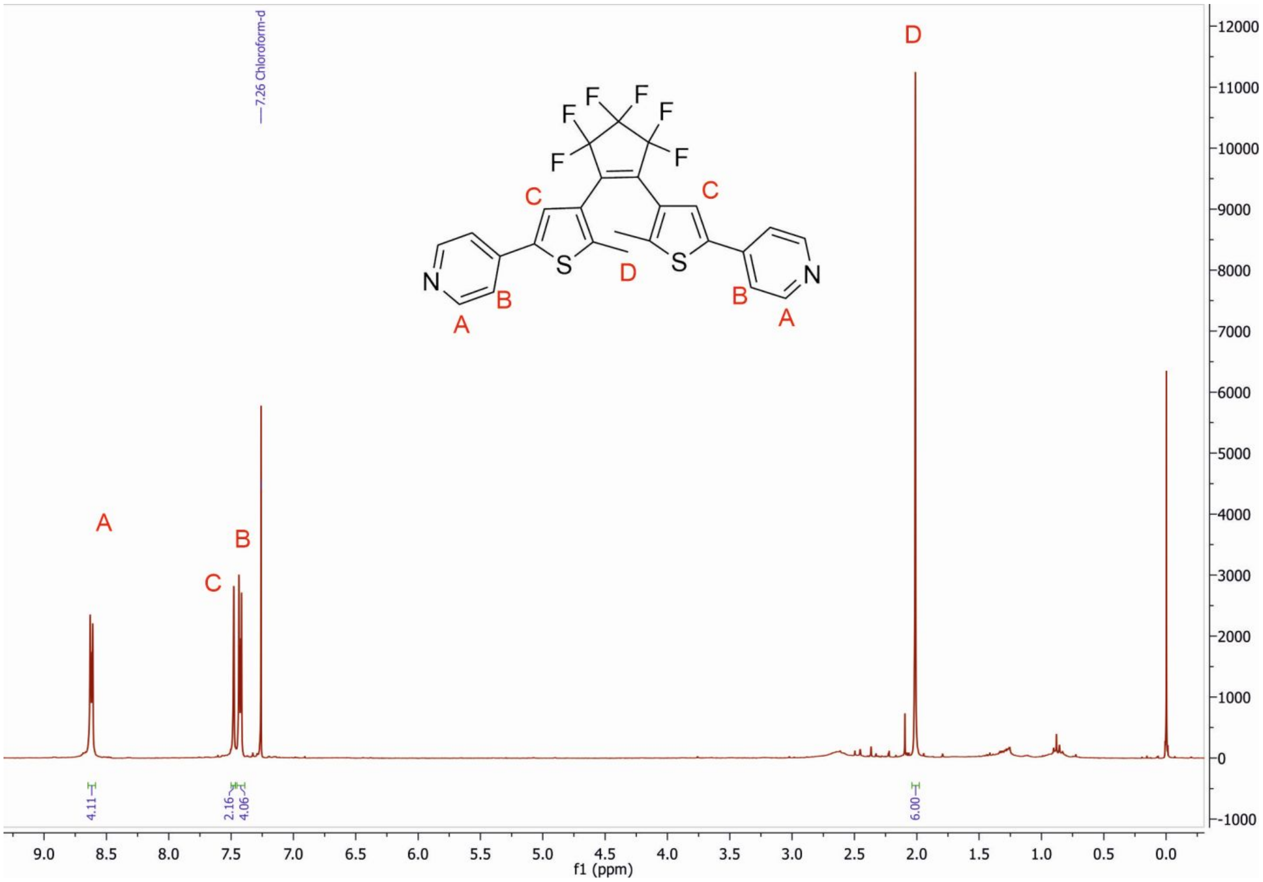

Figure S11. ^{1}H NMR spectrum of 1,2-bis(2'-methyl)-5'-(pyrid-4''-yl)thien-3'-yl)perfluorocyclopentene (dtepy) recorded in $CDCl_3$ with TMS as the reference

References in the Supporting Information:

1. Nikolayenko VI, Castell DC, van Heerden DP, Barbour LJ. Guest-Induced Structural Transformations in a Porous Halogen-Bonded Framework. *Angewandte Chemie International Edition* **57**, 12086-12091 (2018)
2. Valderrey V, Bonasera A, Fredrich S, Hecht S. Light-Activated Sensitive Probes for Amine Detection. *Angewandte Chemie International Edition* **56**, 1914-1918 (2017)
